# Pan-cancer analysis revealing that PTPN2 is an indicator of risk stratification for acute myeloid leukemia

**DOI:** 10.1038/s41598-023-44892-z

**Published:** 2023-10-26

**Authors:** Xuanyu Wang, Sanyun Wu, Le Sun, Peipei Jin, Jianmin Zhang, Wen Liu, Zhuo Zhan, Zisong Wang, Xiaoping Liu, Li He

**Affiliations:** 1 Department of Urology, Zhongnan Hospital of Wuhan University, 169 Donghu Road, Wuhan, 430071 China; 2 Department of Hematology, Zhongnan Hospital of Wuhan University, Wuhan, 430071 China; 3 School of Basic Medical Sciences, Wuhan University, Wuhan, 430071 Hubei Province China; 4 Department of Pathology, Zhongnan Hospital of Wuhan University, 169 Donghu Road, Wuhan, 430071 China

**Keywords:** Leukaemia, Tumour biomarkers, Prognostic markers

## Abstract

The non-receptor protein tyrosine phosphatases gene family (PTPNs) is involved in the tumorigenesis and development of many cancers, but the role of PTPNs in acute myeloid leukemia (AML) remains unclear. After a comprehensive evaluation on the expression patterns and immunological effects of PTPNs using a pan-cancer analysis based on RNA sequencing data obtained from The Cancer Genome Atlas, the most valuable gene PTPN2 was discovered. Further investigation of the expression patterns of PTPN2 in different tissues and cells showed a robust correlation with AML. PTPN2 was then systematically correlated with immunological signatures in the AML tumor microenvironment and its differential expression was verified using clinical samples. In addition, a prediction model, being validated and compared with other models, was developed in our research. The systematic analysis of PTPN family reveals that the effect of PTPNs on cancer may be correlated to mediating cell cycle-related pathways. It was then found that PTPN2 was highly expressed in hematologic diseases and bone marrow tissues, and its differential expression in AML patients and normal humans was verified by clinical samples. Based on its correlation with immune infiltrates, immunomodulators, and immune checkpoint, PTPN2 was found to be a reliable biomarker in the immunotherapy cohort and a prognostic predictor of AML. And PTPN2'riskscore can accurately predict the prognosis and response of cancer immunotherapy. These findings revealed the correlation between PTPNs and immunophenotype, which may be related to cell cycle. PTPN2 was differentially expressed between clinical AML patients and normal people. It is a diagnostic biomarker and potentially therapeutic target, providing targeted guidance for clinical treatment.

## Introduction

Acute myeloid leukemia (AML) is an aggressive and highly heterogeneous disease with different subtypes and distinct clinical outcomes^1^. Recent advances have been made in the targeted therapies of AML. In the future, the combination of targeted drugs with induction chemotherapy will be routine, and targeted drugs will become the standard approach for induction, consolidation and post-consolidation maintenance therapy^2^. Therefore, it is required to identify novel biomarkers and therapeutic targets that are meaningful for the evaluation of the prognosis and treatment response of patients with AML. At the same time, the 5-year survival rate for patients with AML remains dishearteningly low at 28.3%. Furthermore, most cases experience frequent relapses after achieving remission^3,4^. Therefore, a new prognostic model is needed for the risk stratification and treatment guidance of patients.

Intracellular non-receptor PTPs (PTPNs), the largest cysteine PTP family, play an important role in the occurrence, development, metastasis and drug resistance of some types of tumors, and PTPNs inhibitors have significant potential in antitumor therapy^5^. Previous studies have shown that inhibition of PTPN2 is a potential therapeutic strategy to improve the effectiveness of cancer immunotherapy^6^. However, there are still deficiencies in the analysis of PTPN2 at the level of pan-carcinoma, especially its role in AML, which should be further studied.

In the present study, we demonstrated that the role of PTPNs in cancer may be related to mediating cell cycle related pathways, confirmed differential expression of PTPN2 at the clinical level between AML patients and normal subjects, indicated that AML may be a promising candidate for PTPN2 suppression immunotherapy, and constructed a nomogram for risk assessment in AML.

## Results

### Depicting the pan-cancer expression pattern, prognostic value, immune response, drug therapy, and functional assessment of PTPNs

**Figure 1:**
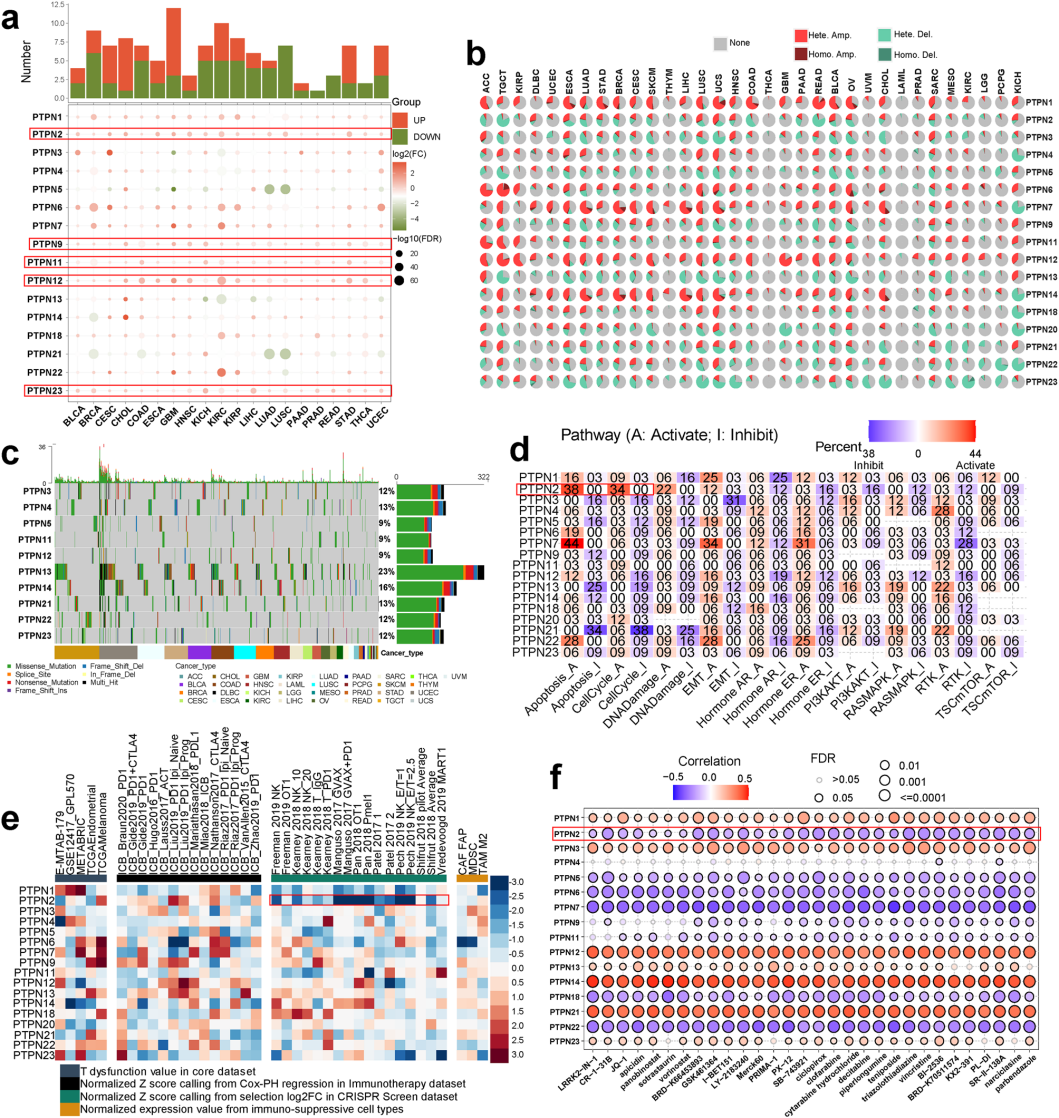
Pan-cancer analysis of PTPNs. (**a**) Differential expression of PTPNs. (**b**) The constitute of the Heterozygous/Homozygous CNV of PTPNs in pan-cancer. (**c**) The mutation distribution of the top 10 mutated genes in PTPNs and a SNV classification of SNV types. (**d**) The percentage of cancers in which PTPNs expression has potential effect (FDR <  = 0.05) on pathway activity. (**e**) Priority of PTPNs among four immunosuppressive indices, including the T-cell dysfunction levels, ICB response outcome, phenotypes in CRISPR screens, and T-cell exclusion cell types. (**f**) Correlation between PTPNs expression and drug IC50.

The expression and prognostic significance of PTPNs in pan-cancer were thoroughly examined. Obvious differences could be found in the expression levels of PTPNs in pan-cancer (Fig. S1a). Then, we obtained transcriptomic data of 20 cancers with both tumorous and normal tissues from the TCGA cohort. Moreover, PTPNs expressions except for PTPN5, PTPN13, and PTPN21 were significantly upregulated in the tumorous compared with those in normal tissues in 20 cancers in the TCGA pan-cancer (Fig. 1a). Furthermore, the prognostic value of PTPNs in pan-cancer was studied. PTPNs exhibited remarkable value in predicting OS, DFI, DSS, and PFI in many tumor types (Fig. S1B). In KIRC, PTPN3 and PTPN7 expression is significantly distinct among pathologic stage (from stage I to IV) (Fig. S1c). In BRCA and KIRC, the expression levels of PTPNs were significantly distinct among subtypes (Fig. S1d).

Single nucleotide variation (SNV) and copy number variation (CNV) are important variations that lead to changes in gene expression during tumorigenesis and tumor growth^7,8^. The waterfall plot showed mutations landscape of PTPNs in 33 types of cancer and the bar chart displays the CNV profile of PTPNs, and provided the classification of CNV types (Fig. 1c), and PTPN13 was the most frequently mutated gene (Fig. S2a). Then, survival analysis of mutant and wild types indicated that mutations of PTPN6 and PTPN23 were risk factors for LIHC, mutations of PTPN1 and PTPN9 were risk factors for AML, and mutations of PTPN14 were risk factors for LUSC (Fig. S2g). The CNV percentage in pan-cancer indicated that heterozygous amplification in cancers were widely found in genes PTPN1, PTPN7, PTPN12 and PTPN14 in most cancers, while heterozygous deletions were widely found in genes PTPN13, PTPN20, PTPN22 and PTPN23 (Fig. 1b). Further investigation indicated positive correlations between CNV and mRNA expressions in most cancers (Fig. S2c). Then, the correlation between CNV and survival time of patients in the pan-cancer was evaluated, which suggested that the most effective gene of LIHC was PTPN6 (Fig. S2f.).

The dysregulation of DNA methylation is closely related to the onset of various diseases, including cancer^9^. The study on the methylation of PTPNs in pan-cancer might help us to reveal the correlations between PTPNs and cancer occurrence to a certain extent. Our analysis indicated significant methylation differences between tumor and normal tissues (Fig. S2d), and negative correlations between DNA methylations and mRNA expressions in most cancers (Fig. S2e). Then, the correlation between methylation and survival index (OS, DSS, DFI, PFS) in pan-cancer was evaluated, and the results indicated that the methylation upregulation of PTPN4 was a risk factor of UVM (Fig. S2h).

Based on the above analysis, PTPNs were likely to be strongly correlated with immunotherapy and drug sensitivity. To confirm this conjecture, the correlations of gene level scores of PTPNs with signatures of T-cell dysfunction, exclusion and CRISPR screen of anticancer immunity were summarized. The results showed that PTPNs were extensively correlated with T-cell dysfunction levels. Notably, significant negative correlations were found between PTPN2 and phenotypes in CRISPR screens. Among the cell types that promoted T cell rejection, both cancer associated fibroblasts (CAFs) and myeloid-derived suppressor cells (MDSC) had significant negative correlations with PTPN6 expression (Fig. 1e). Correlation analysis between PTPNs expression and drug IC50 based on the CTRP database revealed that PTPNs expressions except for PTPN4 were significantly correlated with the responsiveness of most drugs (Fig. 1f) and similar findings were found on the GDSC cohort (Fig. S2j).

To investigate potential mechanisms that PTPNs affect immune response and drug sensitivity, GSEA was performed, and the results indicated that the DEGs were mainly enriched in cell cycle related pathways (Myc target V1, G2M checkpoint, and E2F_targets) (Fig. S2i). Further studies suggested that PTPN7 significantly activated apoptosis, and EMT pathway in pan-cancer, PTPN2 significantly activated apoptosis and cell cycle pathway in pan-cancer, while PTPN21 significantly inhibited apoptosis and cell cycle pathway in pan-cancer (Fig. 1d). These results suggested that PTPNs primarily activated and inhibited apoptotic and cell cycle pathways to participate in the process of tumor proliferation.

### Depicting the pan-cancer expression pattern and genomic pattern of PTPN2

In the above studies, PTPN2 is widely up-regulated of expression and significantly activates apoptosis and cell cycle pathways in pan-cancer. In CRISPR screening, PTPN2 was negatively correlated with phenotype^10^. Considering the high expression of PTPN2 in hematopoietic cell at the same time, a further study on PTPN2 was further carried out.

**Figure 2:**
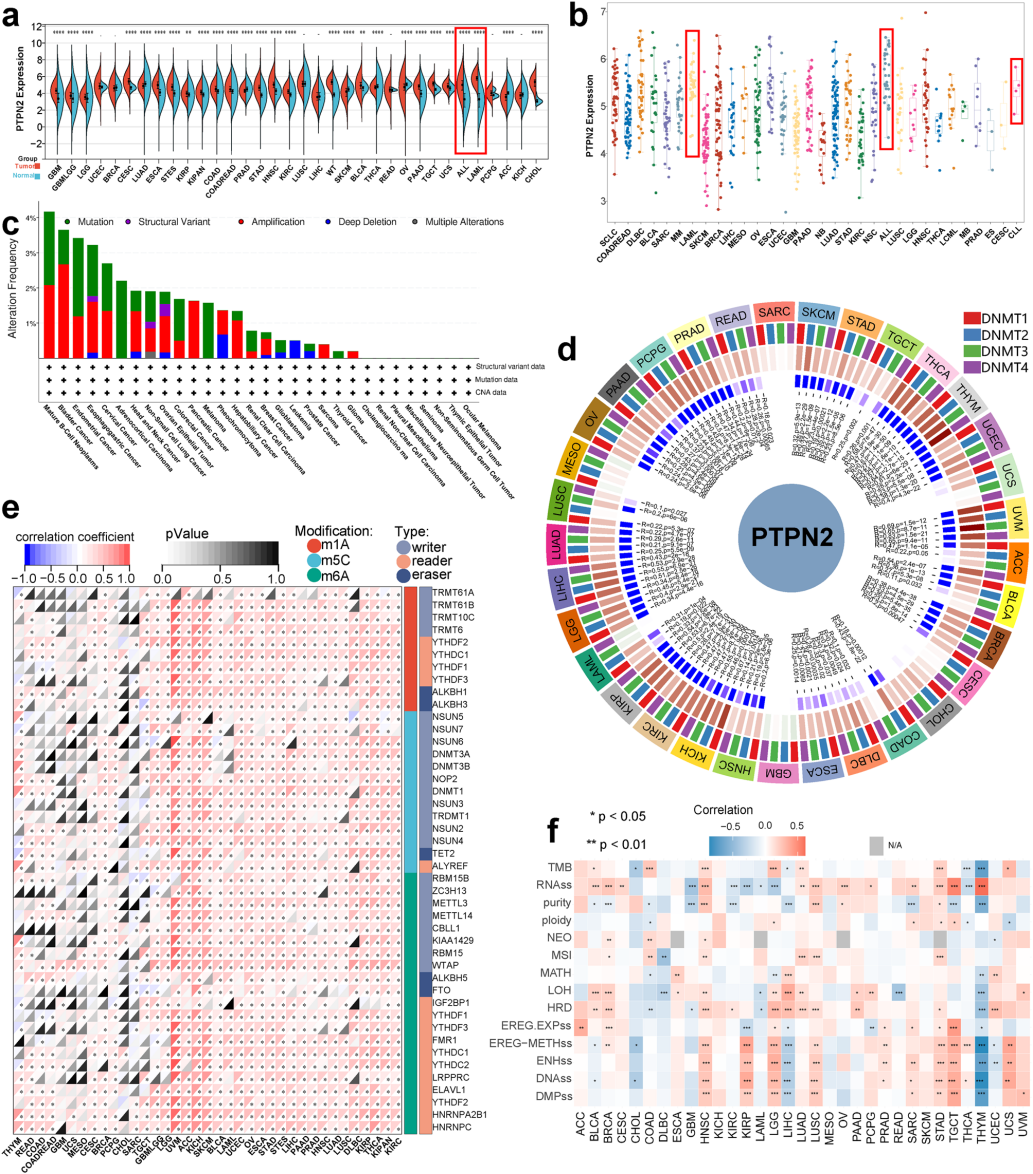
Pan-cancer analysis of PTPN2. (**a**) Expression of PTPN2 in normal and tumor tissues. (**b**) Expression analysis of PTPN2 in pan-cancer. (**c**) Alteration frequency of PTPN2. (**d**–**f**) Correlation between PTPN2 and methyltransferases, modification regulators, stemness score, and tumor heterogeneity. *P < 0.05, **P < 0.01, ***P < 0.001, ****P < 0.0001.

The expression of PTPN2 was significantly up-regulated in several cancers (Fig. 2a), it was significantly expressed in almost all types of cancer, especially LAML, ESCA, ALL, and CLL (Fig. 2b). Moreover, PTPN2 protein was highly expressed in tumor tissues according to the Clinical Proteomic Tumor Analysis Consortium (CPTAC) project (Fig. S3a). This reveals the correlation between PTPN2 and cancer occurrence to a certain extent. Then, The Human Protein Atlas (HPA) database was used to evaluate the RNA and protein expression of PTPN2 in various organs. The results indicated that PTPN2 was highly expressed in immune-related organs such as lymph node, tonsil, bone marrow, and thymus (Fig. S3c). This was consistent with Harmonizome database results, suggesting that the link between PTPN2 and the immune system and blood system was robust. In addition, PTPN2 was almost not expressed in adipose tissue (Fig. S3d).

Next, we evaluated the expression of PTPN2 in paired tumorous and normal samples and the results indicated that PTPN2 was differentially expressed in normal and tumor tissues in 13 types of cancer and was generally upregulated in tumor tissues (Fig. S3b). By obtaining subcellular localization of PTPN2 in the HPA, PTPN2 was found in the nucleoplasm and was highly expressed in almost all cancer cell lines including SiHa, U-2 OS, U-251 MG, A-431, and CACO-2 (Fig. S3e). The expression levels of PTPN2 across cell lines and tissues were analyzed based on the Harmonizome database. The results indicated that PTPN2 was widely expressed in whole body organs. Notably, PTPN2 expression was highest in lymphocytes and blood (Fig. S3f.). Its expression in different tissues of different organs and different systems was analyzed. The results indicated that PTPN2 was widely expressed in immune system, especially in thymus tissues (Fig. S3g).

Broadly speaking, mutation and amplification are the most common mutation types in PTPN2, accounting for all in some cancers (Fig. 2c). Lollipop chart indicated that PTPN2 mutations mainly occurred in Y_phosphatase between 0 and 415 amino acids, with missense as the dominant type of mutation. For different Exons, mutations mainly occurred in Exon 8, and the mutation site S298C/F/Y had the highest mutation frequency (Fig. S4a). Analysis results demonstrated that the type of CNV is correlated with PTPN2 expression. Among these, amplification has the highest mRNA expression level (Fig. S4c). PTPN2 variants remained mostly to be shallow deletions, while the peak of mutation count appeared at the endometrial cancer and melanoma (Fig. S4b). Fraction Genome Altered (FGA) of PTPN2 in 30 types of cancer was detected, and the results indicated that shallow deletions are widespread in many cancer types (Fig. S4d). MMR is an intracellular mismatch repair mechanism, and the loss of function of key genes in this mechanism will lead to the failure of DNA replication errors to be repaired, resulting in a higher rate of somatic mutation, and it’s a potential cancer driver^11^. Therefore, the correlations between the expression of PTPN2 and five important MMRs related genes (MLH1, MSH2, MSH6, PMS2 and EPCAM) in pan-cancer were analyzed, which indicated that PTPN2 was significantly correlated with MMRs genes in 29 types of cancer. In these tumors, MLH1, MSH2, MSH6 and PMS2 were positively correlated with PTPN2, suggesting that PTPN2 may play a role in tumors through the regulation of MMRs process (Fig. S4e).

RNA modification can directly affect the chemistry of RNA and thus affect cancer progression^9^. Therefore, 44 RNA modification regulators of three types of cancer-related RNA modifications were collected, including N6-methyladenosine (m6A), N1-methyladenosine (m1A), and 5-methylcytosine (m5C), and the correlation with PTPN2 expression was analyzed. The results showed that the expression of PTPN2 was significantly positively correlated with RNA modified genes in most cancers. This suggests that PTPN2 expression potentially affects the RNA modification process in pan-cancer (Fig. 2e). The expression of four methylated transferases (DNMT1, DNMT2, DNMT3A and DNMT3B) in various tumor types was significantly correlated with the expression of PTPN2. Notably, the co-expression coefficients of SKCM and STAD were significantly higher (Fig. 2d).

Then we analyzed the correlation between PTPN2 expression and stemness score, and tumor heterogeneity, like RNAss (RNA expression-based), EREG.EXPss (epigenetically regulated RNA expression-based), DNAss (DNA methylation-based), EREG-METHss (epigenetically regulated DNA methylation-based), DMPss (differentially methylated probes-based), ENHss (enhancer Elements/DNA methylation-based), TMB (tumor mutational burden), MATH, MSI (microsatellite instability), purity, ploidy, HRD, LOH, and NEO. We found significant correlations between PTPN2 expression and stemness score, and tumor heterogeneity (Fig. 2f).

### PTPN2 expression is related to ICP, immunomodulatory genes, and immune infiltration levels in pan-cancer

**Figure 3:**
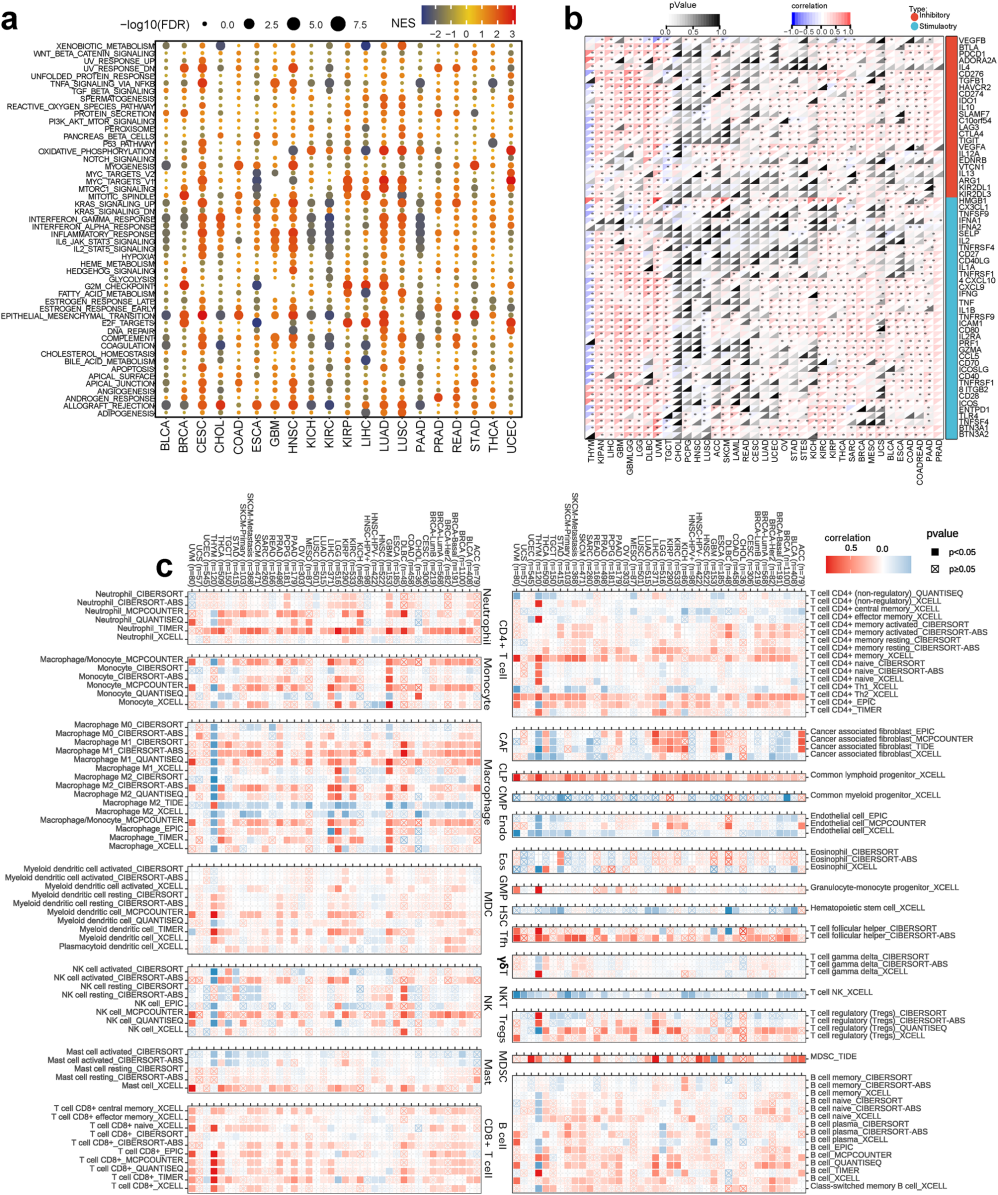
Association of PTPN2 with cancer pathways and immune processes. (**a**) Immunophenotypes Enrichment analysis for metabolism pathway and cancer signaling between high and low PTPN2 expression. (**b**) Correlation between PTPN2 and ICP. (**c**) Correlation between PTPN2 expression and immune infiltration in pan-cancer. *P < 0.05.

To explore the main pathways through which PTPN2 exerts immunomodulatory effects, the samples were grouped according to the PTPN2 expression. Then DEGs among groups were screened and subjected to GSEA to analyze the correlation between PTPN2 expression and cancer-related pathways. The results indicated that the DEGs were mainly enriched in cell cycle-related pathways (MYC, and E2F), Inferon γ response, Inflammatory response, epithelial mesenchymal transition, allograft rejection, and oxidative phosphorylation (Fig. 3a).

Previous studies proved that ICPs were important for maintaining self-tolerance and preventing excessive immune responses that could cause damage to healthy tissues. However, some cancer cells could take advantage of these checkpoints to evade the immune system's attack^12^. Therefore, we investigated the correlations between the expression level of ICPs and PTPN2 in pan-cancer to characterize the potential role of PTPN2 in immunotherapy and the results indicated that the expression of PTPN2 was significantly positively correlated with ICPs in most cancers (Fig. 3b), which suggested that PTPN2 might coordinate the activity of ICPs in different pathways and might be considered an ideal immunotherapeutic marker. We also examined the correlations between the expression of various immunomodulatory genes, such as chemokine receptors, MHC molecules, immune-inhibitors, and immune-stimulators, and PTPN2 expression. The results showed a significant positive correlation between PTPN2 expression and immunomodulatory genes in most types of cancer (Fig. S5a).

We used TIMER2.0 to analyze the correlation between PTPN2 expression and immune cell infiltration in pan-cancer. The results showed a positive correlation between PTPN2 expression and various immune infiltrates, including common lymphoid progenitor, T cell follicular helper, myeloid-derived suppressor cells, B cell, neutrophil, monocyte, macrophage, myeloid dendritic cell, and CD8^+^ T cell, and a negative correlation with common myeloid progenitor, endothelial cell, hematopoietic stem cell, and NKT cell (Fig. 3c). PTPN2 was found to be involved in immune infiltration and played an important role in immune-tumor interaction. It should be noted that the trend of this correlation was different in THYM, especially B cell and Macrophage, which may be correlated to the different tumor microenvironment^13^. PTPN2 was also found to be widely correlated with ESTIMATE score, immune score, and stromal score in pan-cancer (Fig. S5b–d) The findings indicated that PTPN2 played a vital role in immune infiltrates in pan-cancer and has the potential to serve as a response indicator in clinical practice.

### Exploring the immunotherapy response, prognostic correlation, drug sensitivity, and predictive power of PTPN2 in pan-cancer

**Figure 4:**
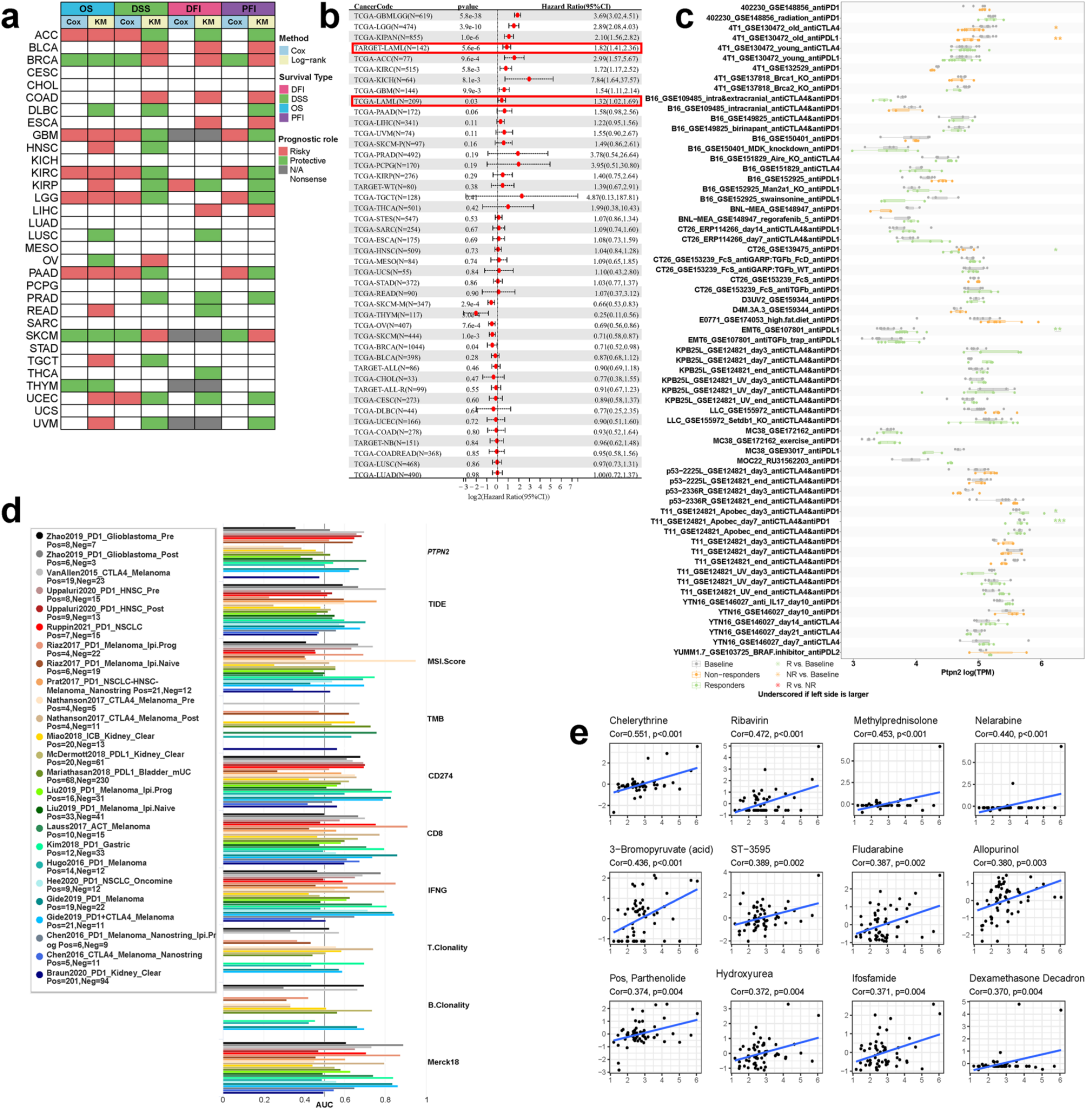
Prognostic value and biomarker potential of PTPN2. (**a**) Effect of PTPN2 on cancer prognosis. (**b**) Correlation between PTPN2 expression and overall survival in the TCGA and TARGET cohort. (**c**) A Comparison of PTPN2 expression before and after ICB treatments across different tumor models in vivo. (**d**) Ability of PTPN2 to predict response outcome and overall survival in immunotherapy cohorts. (**e**) The top 12 drugs positively correlated with PTPN2 expression in the CellMiner database.

We collected survival data from TCGA and TARGET data portals to evaluate the prognostic value of PTPN2 in pan-cancer using CoxPH and log-rank test. PTPN2 expression was found to be a reliable biomarker in a wide range of cancer types, and was significantly correlated with overall survival in AML (Fig. 4a,b; Fig. S6a–c).

To explore the promising value of PTPN2 as a novel immune target, the immunotherapy response and sensitive drugs among different PTPN2 expression were compared. The results revealed that there were significant differences in PTPN2 expression among 12 murine immunotherapy cohorts (Fig. S6d). Among them, IFN-γ or TNF-α treated mice were more likely to have elevated PTPN2 levels, while TGF-β1 treated mice were more likely to have low expression of PTPN2. The results also revealed that PTPN2 could significantly predict immunotherapy response in 5 murine immunotherapy cohorts, which responders were more likely to have elevated PTPN2 levels (Fig. 4c). Additionally, PTPN2 was closely correlated to the efficacy of immunotherapy such as CAR-T, PD-L1, Anti-PD-1 and Anti-CTLA-4, indicating the potential of PTPN as an immunotherapy biomarker (Fig. S7).

Then, comparisons between PTPN2 expression and other published biomarkers based on their predictive power of immunotherapy response were performed, it was found that PTPN2 had an AUC of more than 0.5 in 12 of the 25 immunotherapy cohorts (Fig. 4d). Compared with TMB (AUC > 0.5 in 8 cohorts), T.Clonality (AUC > 0.5 in 9 cohorts), and B.Clonality (AUC > 0.5 in 7 cohorts), PTPN2 exhibited a higher predictive value. These results demonstrated the promising value of PTPN2 as a biomarker.

The positive (Fig. 4e) and negative (Fig. S6e) correlation between drug sensitivity and PTPN2 expression was analyzed. The data suggested that PTPN2 might be correlated with chemical resistance to some commonly used antitumor drugs in clinical practice, such as Nelarabine, Fludarabine and Hydroxyurea. Among them, Nelarabine and Fludarabine can have certain curative effect on leukemia, suggesting that PTPN2 is closely correlated to drug resistance in patients with hematologic tumors.

### Elucidating the correlation between PTPN2 and microenvironment in AML

**Figure 5:**
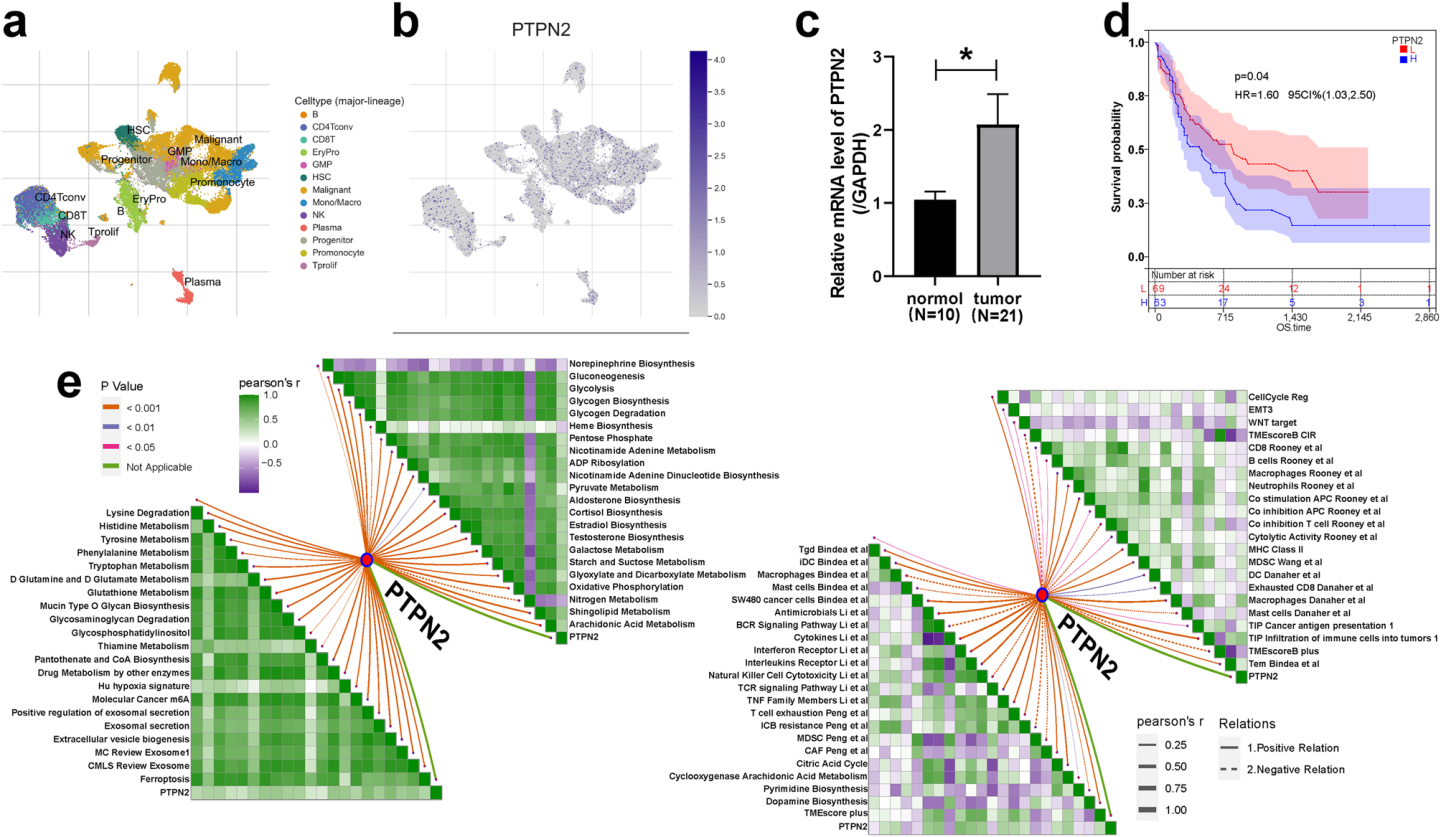
Prognostic value and biomarker potential of PTPN2. (**a**, **b**) The expression of PTPN2 in different cell types. (**c**) Expression level of PTPN2 in the bone marrow samples of AML patients and normal donors. (**d**) The prognostic role of PTPN2 in the TCGA-LAML cohort. (**e**, **f**) Correlation between PTPN2 expression and cancer microenvironment-related signatures in TCGA-LAML. *P < 0.05, **P < 0.01, ***P < 0.001.

In the above studies, potential correlations between PTPN2 and hematological diseases were found, thus, it is necessary to further explore the expression pattern of PTPN2 in AML. PTPN2 was differentially expressed in different cell species in GSE116256 (Fig. 5a, b), GSE135851, and GSE154109 (Fig. S9). The results indicated that PTPN2 was highly expressed in CD8^+^ Tex cell, plasma cell, monocyte and promonocyte, and low expressed in CD4^+^ T cell and hepatic stellate cell. Moreover, PTPN2 was strongly correlated with Immune response (Fig. S8a). Results of qRT-PCR confirmed the expression level of PTPN2 in the bone marrow samples of 21 AML patients was significantly higher than that of 10 normal donors (Fig. 5c). The prognostic role of PTPN2 in the TCGA-LAML cohort was also investigated, suggesting that PTPN2 is a diagnostic biomarker for AML and may also be a prognostic marker (Fig. 5d). We collected 87 signatures related to TME and tumor phenotypes from the IOBR package and analyzed the correlation between PTPN2 expression and these signatures. The results showed that PTPN2 expression had extensive and consistent positive or negative correlations with the signatures in TCGA (Fig. S8b,c) and beatAML (Fig. 5e,f) cohorts, indicating its close association with TME.

### Drug discovery of PTPN2 in AML

**Figure 6:**
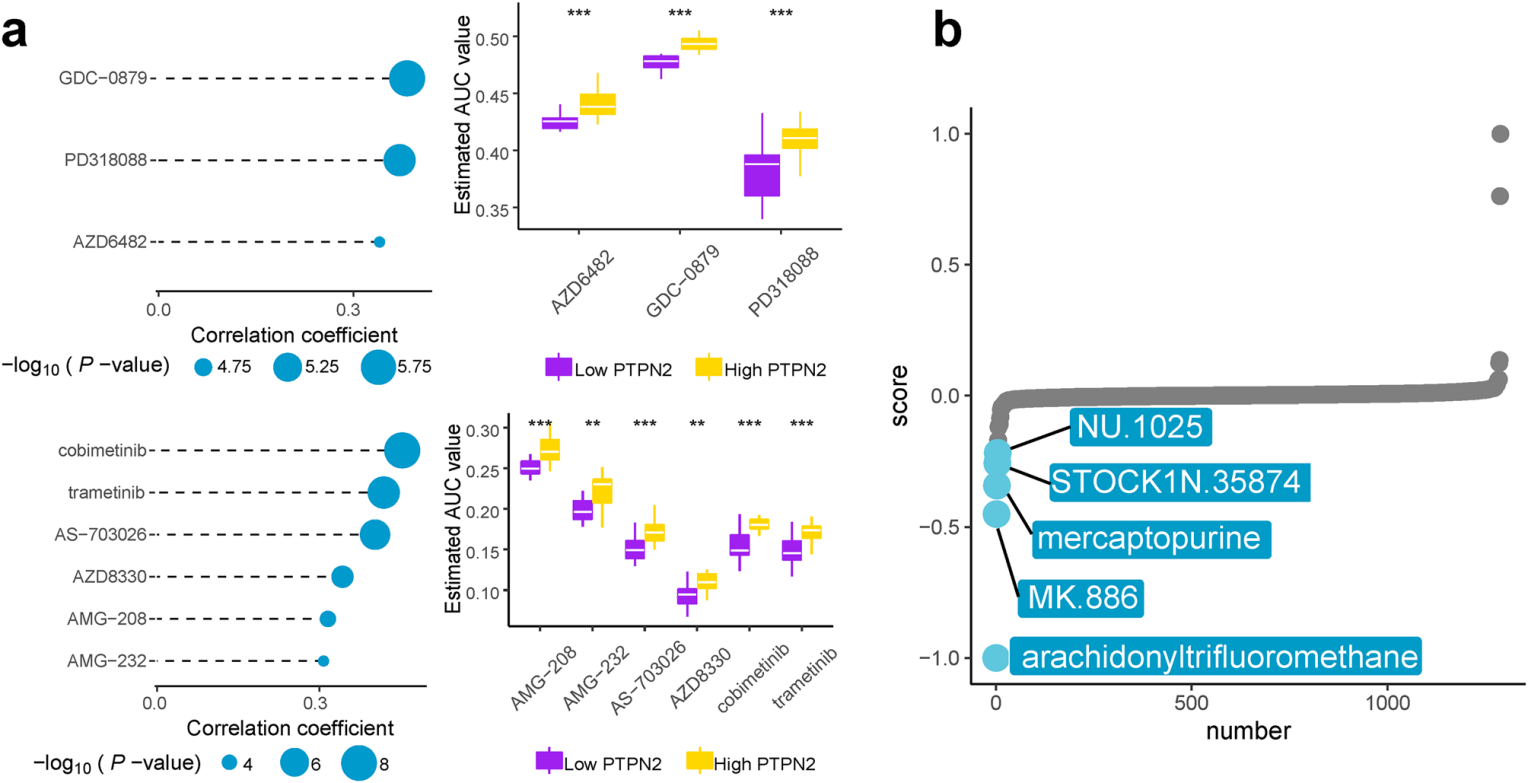
Drug discovery of PTPN2 in AML. (**a**) Drug screening in patients with high PTPN2 expression in CTRP and PRISM. (**b**) CMAP analysis between high and low PTPN2 expression groups.

The drug sensitivity of data, obtained from CCLE in CTRP and the PRISM, indicated that patients with low PTPN2 expression were highly sensitive to three CTRP-derived compounds and six PRISM-derived compounds (Fig. 6a), high PTPN2 expression were highly sensitive to five CTRP-derived compounds (panobinostat, ouabain, neuronal differentiation inducer III, BRD-K61166597 and B02) and three PRISM-derived compounds (romidepsin, RGFP966, and imidapril) (Fig. S10). After that, the difference between high and low PTPN2 expression groups was analyzed to obtain the molecular characteristics of the disease for cMAP analysis. Results showed five compounds were identified to be mostly correlated with PTPN2 expression characteristics (Fig. 6b). Among them, mercaptopurine was often used as a therapeutic agent for leukemia, indicating that the correlation between PTPN2 and hematological cancers was robust.

### Derivation, construction and validation and characterization of PTPRS

**Figure 7:**
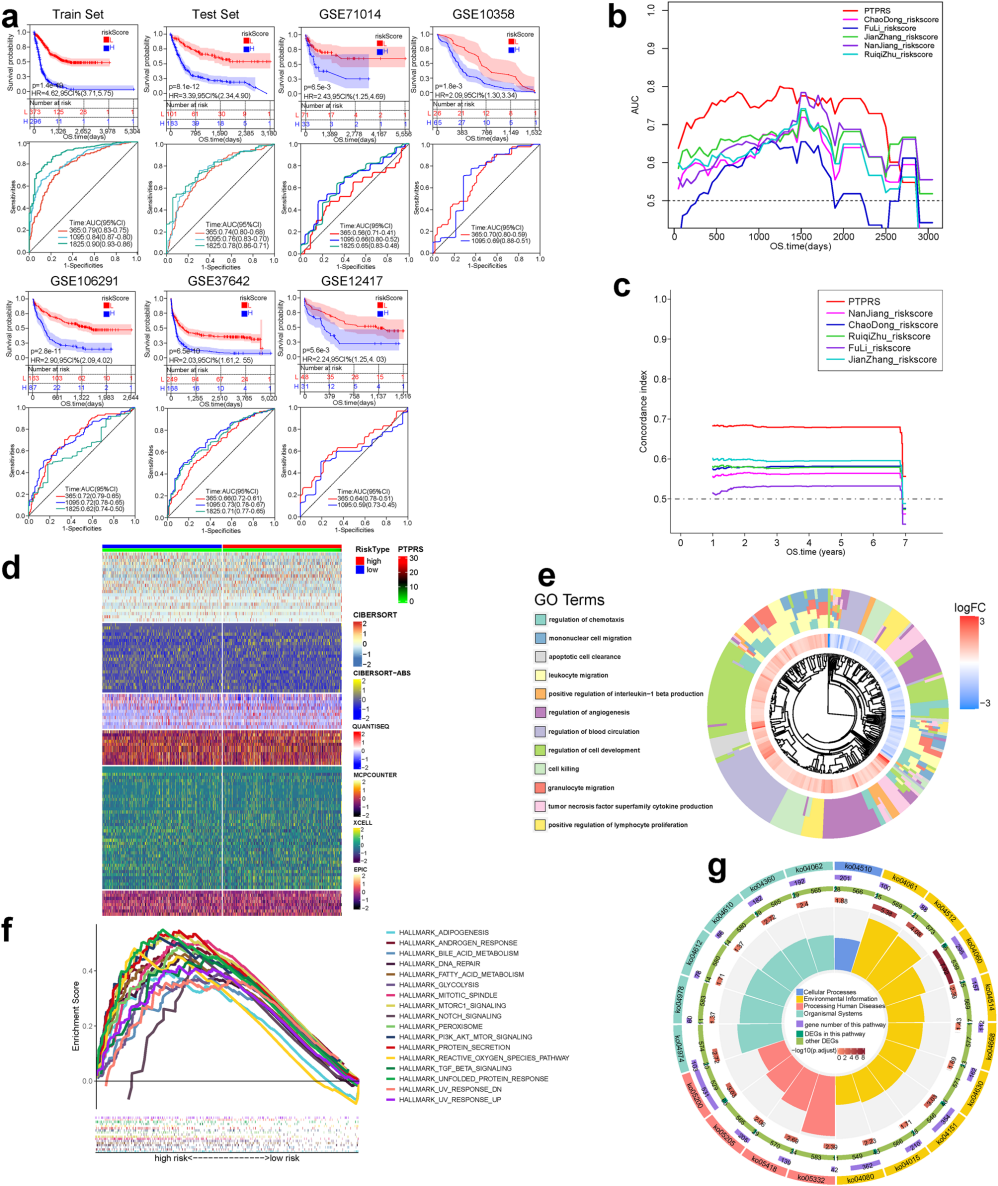
Construction and characterization of PTPRS. (**a**) Development of PTPRS in metaX training set and the predictive accuracy of PTPRS for survival. Validation of the PTPRS in metaX validation set and five external independent sets, including GSE71014, GSE37642, GSE106291, GSE12417 and GSE10358. (**b**, **c**) ROC-AUC value and C-index in different risk scoring systems. (**d**) Heatmap for infiltration of immune based on CIBERSORT, CIBERSORT-ABS, QUANTISEQ, MCPCOUNTER, XCELL, and EPIC algorithms among high-risk and low-risk group. (**e**–**g**) Functional and signaling pathway analysis of these differential genes in AML according to GO, Hallmarks, and KEGG pathway. *P < 0.05, **P < 0.01, ***P < 0.001.

The study found that PTPN2 expression in AML had a significant effect on biological characteristics and clinical outcomes. Due to the complexity of PTPN2 expression, a PTPRS was constructed to approximate and simplify it. We formed metaX cohorts (n = 953) and metaY cohorts (n = 771) as mentioned above, and then 953 samples in the metaX cohort were randomly grouped into training set (n = 669) and validation set (n = 284) in a ratio of 7:3. Using Cor.test function, 8667 PTPN2 related genes in metaX and 5081 genes in metaY were screened, and 1824 genes were obtained by intersection. Then, in the training set, 826 prognostic related genes were screened out by Kaplan–Meier analysis, 30 of which were selected as effective candidate genes by univariate and LASSO CoxPH model, and finally 24 robust genes were screened out by stepwise multivariate CoxPH to construct PTPRS (Fig. S11a,b; Table S3). In the training group, patients in the training set, validation set, and other external validation cohorts were grouped into high-risk and low-risk groups based on the median of score. Patients in the low-risk groups had longer survival time than those in the high-risk groups, suggesting that PTPRS is a reliable prognostic indicator in all AML cohorts (Fig. 7a). To test the reliability of the PTPRS, patients in TARGET-AML and TCGA-LAML were combined into overall CP cohort (n = 516) and the prediction abilities of the PTPRS and five existing prediction systems were compared, and the results suggested that PTPRS has better prediction ability (Fig. 7b,c). In addition, results of univariate (Fig. S11c) and multivariate CoxPH (Fig. S11d) in the TCGA-LAML and TARGET-AML cohort suggested that PTPRS remained a significantly and independently prognostic factor after adjusting for other clinical factors. Taken together, these results validated the good prognostic efficiency of PTPRS.

75 immunomodulatory genes were collected to analyze their expression, methylation and mutation characteristics in high-risk and low-risk groups. In the high-risk group, immunomodulatory genes expression had a more significant positive correlation with methylation level; and in the low-risk group, immunomodulatory genes amplification and deletion frequency was higher (Fig. S11e). At the same time, it was found that the high-risk group had higher immune checkpoint target-related gene expression, immune score, and immune cell infiltration levels (Fig. S11f.). Based on different immune cell algorithms, the high-risk and low-risk group had different immune infiltration conditions, indicating that the high-risk and low-risk group may had different immune microenvironments (Fig. 7d). We analyzed infiltration levels in 27 cells in the high-risk and low-risk group. The result indicated that high-risk group had significantly higher levels of immune cell infiltration (Fig. S11g). The high and low risk groups had different mutation frequencies. Except for RUNX1, IDH2, and KRAS, the high-risk group had lower mutation frequencies than the low-risk group (Fig. S11h).

By applying GO and KEGG pathway enrichment analysis, the results indicated that biological processes (Fig. 7e) were mainly enriched in cell migration and cell cycle related pathways, cellular components (Fig. S11i) were mainly enriched in ribosome, lysosome and respiratory chain related pathways, and molecular function (Fig. S11j) was mainly enriched in cytokine and immune receptor related pathways. DEGs were significantly enriched in four KEGG terms (Graft-versus-host disease, Viral protein interaction with cytokine and cytokine receptor, ECM-receptor interaction, and Cytokine-cytokine receptor interaction) (Fig. 7f). To further investigate the potential differences between high-risk and low-risk groups, GSEA enrichment analysis was performed for differential genes. The results indicated that the high-risk group was significantly enriched in cancer-related signaling pathways (Fig. 7g).

### Clinical values of PTPRS and construction of nomogram

**Figure 8:**
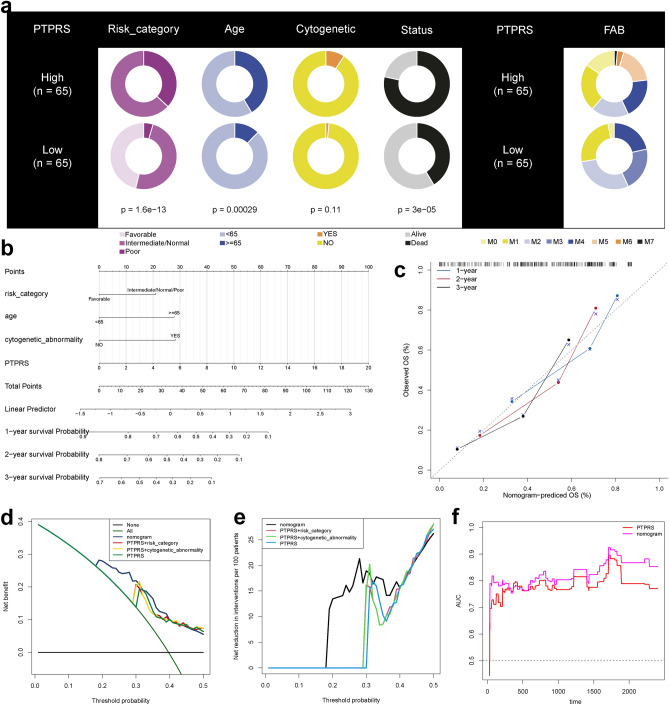
Clinical association of PTPRS and construction of nomogram. (**a**) The difference in clinicopathologic features and pathological stages of AML between high-risk and low-risk group. (**b**) Nomogram predicting the 1-, 2-, and 3-year OS in patients with AML. (**c**) The calibration curves for predicting patient OS at a 1-, 2-, and 3-year. (**d**, **e**) DCA curves of the nomogram, PTPRS and other pooled models for predicting 1-, 2-, and 3-year OS. (**f**) Time-dependent AUC values of nomogram and PTPRS for the prediction of OS.

The risk category in the high-risk group tended to be Poor (p < 0.001), generally older (p < 0.001), and had a worse prognosis (p < 0.001), while there was no statistical difference in cytogenetic abnormality. And there were significant differences in pathological stage between the high-risk and low-risk group (Fig. 8a, S12a). Among 37 cancer drugs, the high-risk group had a lower IC50 value, indicating a higher sensitivity to the drug (Fig. S12b).

To further optimize the prediction effect of PTPRS, a nomogram containing important predictors in CoxPH was established to predict the prognosis of AML (Fig. 8b). For example, patients with AML had a risk category of favorable (0 points), an age of 66 years old (28 points), no cytogenetic abnormalities (0 points), and a PTPRS of 4 (20 points). Therefore, with a total score of 48, the 1-year survival rate is about 49%, 2-year survival rate is about 31%, and 3-year survival rate is about 7%, respectively. Calibration curves showed good agreement between the predicted and observed OS at 1-, 2-, and 3-year in the training and validation cohorts (Fig. 8c). DCA was performed to compare the clinical applicability of the nomogram with PTPRS. Result indicated that the nomogram could better predict OS at 1-, 2-, and 3-year because it added more net clinical benefit compared to PTPRS and other pooled models (Fig. 8d,e). The time dependent AUC curve of OS state was plotted. Changes in AUC over time indicated that nomogram was slightly better than PTPRS in predicting prognosis (Fig. 8f).

## Discussion

Intracellular non-receptor PTPs, the largest of the cysteine PTP family, are critical for the regulation of a variety of biological processes, including but not limited to hematopoietic, inflammatory response, immune system and glucose homeostasis, and play an important role in the occurrence, development, metastasis and drug resistance of tumors^5^. However, comprehensive analysis of PTPNs is still missing at the pan-cancer level, especially in AML, and most studies focus on proving the clinical value of PTPN1 expression and PTPN11 mutation in AML^14,15^. It was found that PTPN2 is highly expressed in hematopoietic cells and plays a negative signaling role^16^. Noteworthily, the PTPN2 catalytic domain shared 74% sequence homology and similar enzyme kinetics with another family member, PTPN1^6^. These results suggest that PTPN2 may play an important role in AML. In this study, the expression and mutation status of PTPNs in pan-cancer were analyzed, finding that PTPN2 is working as a driver of AML. The study also revealed a strong effect of PTPNs on cell cycle and verified PTPN2 as a diagnostic biomarker for patients with AML at clinic level. Finally, PTPRS was developed to predict the prognosis and response of cancer immunotherapy, and a nomogram with better efficacy was constructed combined with clinical indicators.

Targeting PTPNs has always been a crucial approach for treating diseases. According to reports, PTPN1 is involved in the development of many diseases, including obesity, diabetes, cancer, and cardiovascular disease^17^. PTPN1 and PTPN2 inhibitors have been developed and have become emerging means to enhance T cell anti-tumor immunity^18^. PTPN3 is a potential immune checkpoint inhibitor target that may mediate T cells, while PTPN5 and PTPN7 can specifically inactivate MAPKs, so the developed inhibitors may have therapeutic potential for treating neurodegenerative diseases in AML patients^19,20^. Targeting PTPN6 is an effective treatment for combating diabetes^21^. PTPN11 has always been a focus of attention in the field of human diseases, especially cancer, and can bind to multiple immune inhibitory receptors and inhibit the activation of immune cells^22^. PTPN11 regulates numerous cascade pathways, such as RAS-RAF-ERK, JAK-STAT, JAK-STAT, and is closely associated with immunotherapy response^23^. PTPN12 is considered a promising therapeutic target for critical diseases such as cancer, diabetes, metabolic diseases, and autoimmune diseases and has been used for therapeutic intervention in acute myocardial infarction^24,25^. PTPN13 and PTPN23 act as tumor suppressors in various tumors^26–29^. PTPN22 inhibitors have enormous potential to enhance the efficacy of current immunotherapy strategies^30^. However, there are still gaps in the development of targeted drugs for PTPN13, PTPN14, PTPN18, PTPN21, and PTPN23.

Immunotherapy was first identified as an effective treatment for tumors by Wilhelm Bush and Friedrich Fehleisen in the nineteenth century^31^. In recent years, monoclonal antibodies targeting specific targets on tumor cells have been widely used to treat hematological malignancies, either in combination with chemotherapy or as a single agent^32^. PTPN11 is an effective target for the treatment of hematological malignancies and can also bind to various immune inhibitory receptors^22,33^. Considering that PTPN2 and PTPN11 belong to the same family of PTPs, the combination of PTPN2 inhibitors with immune therapy is a promising strategy. The role of PTPN2 as a biomarker in tumor microenvironments was systematically studied. It was found that PTPN2 was strongly correlated to six tumor stemness indexes in many cancers. PTPN2 and RNA modification regulators, ICP, immunomodulatory genes, and mismatch repair related genes were positively correlated.

But in some studies, the absence of PTPN2 in B16 tumours does not produce significant differences in bone marrow cell infiltration^10^. This may be related to the different tumor microenvironments of melanoma and AML, suggesting that PTPN2 may not act as a therapeutic target in all hematologic tumors, but its role in AML is indispensable.

The use of public databases and computational models to identify optimal personalized therapeutic agents and drug combinations has become increasingly popular^34^. In this research, the biomarker correlation and predictive power of PTPN2 in 25 immunotherapy cohorts were analyzed. At the same time, predictions for sensitive drugs have been made based on PTPN2 expression in multiple databases. We found that in 12 immunotherapy cohorts, PTPN2 alone had an AUC of over 0.5, with a higher predictive value than TMB, T.lonality, and B.lonality in the immunotherapy cohort. More importantly, the differential expression of PTPN2 was verified in the bone marrow of patients and normal subjects using clinical samples, and a series of targeted small molecule drugs with good therapeutic effects are predicted in this paper, providing guidance for clinical drug use. Noteworthily, PTPN2 inhibitors have been successfully developed^6^, so the application of PTPN2 inhibitors combined with Immunotherapy in AML has promising potential.

Finally, PTPRS was developed and validated, which is common-used and productive in external validation queues. It has the advantage of combining multiple AML high-throughput sequencing cohorts, and clinical indicators were combined with PTPRS to establish a nomogram with better predictive power.

There are still some limitations to the study. First, although this study largely corrected batch effect across multiple cohorts, the implications for genomic analysis should be further analyzed using larger data sets from multiple databases. Secondly, it is still required to verify the effect of PTPNs on the cell cycle by further experiments. Finally, the predictive effectiveness of PTPRS is not validated in self-tested cohorts.

## Methods

### Data retrieval, collection, and preprocessing

Firstly, The pan-cancer RNA sequencing (RNA-seq) data (FPKM value) and the corresponding survival information of The Cancer Genome Atlas (TCGA)^35^ were extracted from the UCSC Xena Browser (https://xena.ucsc.edu/)^36^. Full names and abbreviations of all cancers are listed in Table S1.

Next, transcriptome information of 151 patients in the TCGA-LAML cohort, 187 patients in the TARGET-AML^37^, 450 patients in the beatAML^38^ cohort (FPKM and Counts value), accompanied by corresponding phenotype and DNA methylation data, were simultaneously downloaded. The copy number variation (CNV) of TCGA-LAML was gathered and processed using the GISTIC 2.0 algorithm^39^, and somatic mutation profiles (Varscan) was obtained as the mutation annotation format (MAF) format by the R package “maftools”^40^.

Then, gene expression profiles and clinical information of GSE10358^41^, GSE12417^42^, GSE23312^43^, GSE32246^44^, GSE37642^45^, GSE71014^46^, and GSE106291^47^ were downloaded from gene expression omnibus (GEO)^48^. Additionally, the expression data of PTPNs in normal cell lines or tissues were retrieved from the BioGPS data portal (http://biogps.org/#goto=welcome)^49^ and Genotype-Tissue Expression (GTEx: https://www.gtexportal.org/home/) project^50^, while expression data of PTPNs in cancer cell lines were downloaded from BioGPS and Cancer Cell Line Encyclopedia (CCLE: https://sites.broadinstitute.org/ccle/) project^51^.

For the TCGA-LAML, TARGET-AML, and beatAML cohorts, the FPKM values were converted into TPM values for consistency, and further subjected to log2(x + 1) transformation for normalization. For all cohorts obtained from the GEO, “normalizeBetweenArrays” function in the R package “limma” was used for normalization^52^.

Next, the abovementioned cohorts were integrated into two AML meta cohorts after excluding patients whose survival information was not available: metaX (n = 953: GSE106291, TARGET, TCGA-LAML, beatAML), metaY (n = 771: GSE12417, GSE23312, GSE32246, GSE37642, GSE71014).

Then, the batch effect was corrected with the “removeBatchEffect” function in “limma”^52^, and their gene expression data was ultimately standardized via Min–Max normalization for downstream multi-database analysis.

### Deciphering pan-cancer expression pattern of PTPNs

We systematically analyzed the expression levels of PTPNs (protein tyrosine phosphatases) between tumorous and adjacent normal tissues at the pan-cancer level using the ONCOMINE^53^, TIMER^54^ and the TCGA-Pan-Cancer atlas.

Furthermore, we conducted further analysis of the expression of PTPN2 protein at the pan-cancer level using the UALCAN platform (http://ualcan.path.uab.edu/index.html), with the Clinical Proteomic Tumor Analysis Consortium (CPTAC: https://proteomics.cancer.gov/programs/cptac) database as a resource^55,56^.

Additionally, we investigated the mRNA and protein levels of PTPN2 expression in normal or tumor tissues and normal or tumor cell lines using the CCLE, Human Protein Atlas (HPA: https://www.proteinatlas.org/), harmonizome (https://maayanlab.cloud/Harmonizome/), and BioGPS portal^57,58^. Furthermore, we deciphered the pan-cancer expression pattern of PTPN2 in the single-cell level using TISCH^59,60^. We utilized the cBioPortal (https://www.cbioportal.org/) to depict the pan-cancer genomic landscape of PTPNs in terms of CNV and single nucleotide polymorphisms (SNPs)^61^. The role of PTPNs in diseases, systematic drug-target identification and prioritization was analyzed preliminarily based on underlying evidence through the Open Targets Platform (https://www.opentargets.org/)^62^. Thanks to GSCA platform for visualizing and demystifying the pan-cancer phenotypic characteristics of PTPNs, this study analyzed the correlation between PTPNs and prognosis or clinical subtypes at the pan-cancer level^63^. Additionally, the relationship between PTPN2 expression and clinical outcomes, including overall survival (OS), progression-free interval (PFI), disease-free interval (DFI) and disease-specific survival (DSS), was analyzed and visualized at the pan-cancer level with the help of Sangerbox^64^ Platform (https://vip.sangerbox.com/home.html)^64^.

### Illustrating pan-cancer biological mechanisms, immunological features, and predictive effect of PTPN2

We performed various analysis to characterize the correlations between PTPN2 expression and multiple molecular features in pans-cancers. These included estimating tumor mutation burden (TMB) and mutant-allele tumor heterogeneity (MATH) using the "maftools" R package^10^, obtaining microsatellite instability (MSI), neoantigen, purity, ploidy, homologous recombination deficiency (HRD), and loss of heterozygosity (LOH) scores^65,66^ and tumor stemness indexes^67^ (RNAss, Ereg. EXPSS, DNAss, EREG-METHss, DMPss, ENHss) from previous studies, and analyzing correlations between PTPN2 and RNA modifier regulators, MMRs related genes, methyltransferases, ICP inhibitors, immunomodulators, and tumor-related signatures at the pan-cancer transcriptome level using Spearman's correlation. Gene set enrichment analysis (GSEA) were performed based on PTPN2 expression (top 30% and bottom 30%) to predict the potential cancer hallmarks related with PTPN2^68,69^. Immunological features of each sample were evaluated using ESTIMATE and immunedeconv algorithms^70,71^. Correlations between PTPN2 and 60 ICPs (24 inhibitory, 36 stimulatory), 150 immunomodulators (41 chemokines, 18 chemokine receptors, 21 MHC molecules, 24 immuno-inhibitors, 46 immuno-stimulators) were illustrated at the pan-cancer transcriptome level (Spearman's Correlation). Tumor immune dysfunction and exclusion (TIDE) and tumor immune syngeneic mouse (TISMO) databases were used to predict immunotherapy and gene treatment responses^72,73^. Correlations between 87 tumor-related signatures and PTPN2 were illustrated using “IOBR” package^74^. Moreover, the intratumoral mutation landscape between the high and low PTPN2 expression groups in the TCGA-LAML cohort was delineated, and Over Representation Analysis and GSEA on KEGG pathways and GO terms were performed^75,76^.

Integrated cell line datasets with drug sensitivity information were extracted from the Genomics of Drug Sensitivity in Cancer (GDSC)^77^, and predicted sensitivity of chemotherapeutic treatment was inferred using oncoPredict^78^. The Cancer Therapeutics Response Portal (CTRP)^79^ and profiling of relative inhibition simultaneously in mixtures (PRISM) database^80^ were used to analyze drug sensitivity relationships between PTPN2 and chemotherapeutic agents. Special thanks are given to Chen Yang for his support with R-script design and splendid analysis methodologies^81^, and Paul Geeleher for development of the R package “pRRophetic”^82^.

Correlation between PTPN2 and drug sensitivity was also analyzed using CellMiner, and CMap score was calculated to predict potential drugs reversing the molecular features of the disease^83–85^.Additionally, PTPN2 expression was analyzed between response and non-response groups in cohorts receiving immunotherapy.

### RNA extraction and qRT-PCR

Total RNA was extracted from bone marrow samples from AML patients and normal individuals using Trizol reagent (Invitrogen, Carlsbad, CA, U.S.A.) according to the manufacturer's protocol. Superscript II reverse transcriptase and random primers were used to synthesize cDNA. Quantitative real-time PCR (qRT-PCR) was performed on the ABI 7900HT Sequence Detection System with SYBR-Green dye (Applied Biosystems, Foster City, CA, U.S.A.). All primers are listed in Table S2. Expression levels of PTPN2 were calculated using the 2-ΔΔCT method.

### Construction and validation of the prediction model

Least absolute shrinkage and selection operator (LASSO) penalized Cox proportional hazards regression model (CoxPH)^86^ with tenfold cross-validation was used to construct a PTPRS for the prognostication of patients^87^. The PTPRS for individual patients was calculated as follows: (gene's expression × coefficient). Univariate and multivariate CoxPHs were used to evaluate the prognostic value of PTPRS. To further unravel the underlying biological mechanisms relating to the PTPRS, differentially expressed genes (DEGs) between different risk groups was screened using DESeq2 package^88^. Functional enrichment analyses (GO and KEGG pathways) as well as GSEA on the DEGs were conducted and visualized via the R package "clusterProfiler 4.0"^89^ and “GOplot”^90^. Kaplan–Meier curves, time-dependent receiver operating characteristic curve (ROC) analysis, decision curve analysis (DCA) and concordance index (C-index) curves were used to evaluate the prognostic role the DEGs by the R package “pROC” and “pec”^91^. Finally, we compared the prediction ability of the PTPRS with other five prognostic^92–96^ models, but also confirmed as an independent prognostic factor in contrast to other clinical biomarkers via multivariate CoxPH.

### Underlying microenvironment between samples in high- and low-risk group

The immune profile was visualized via heatmap, displaying expression of ICP, abundance of 24 immunocyte infiltration, immune score, stromal score and DNA methylation of tumor-infiltrating lymphocytes (MeTILs)^97^.

Then, the differences of 75 immunomodulators between two subtypes were further analyzed at the multi-omics level: mRNA expression, gene expression correlation with DNA-methylation beta-value, amplification frequency (the difference between the fraction of samples in which an immunomodulator was amplified in a particular subtype and the amplification fraction in all samples) and deletion frequency.

Finally, ssGSEA algorithm, which enabled us to quantify the absolute enrichment of various TME infiltration cells via the immune deconvolution analyses, was implemented to investigate the differences of 34 immunocytes in distinct subtypes.

### Establishment, superiority and validation of a nomogram speculating prognosis

To further quantify the predictive performance of PTPRS, we constructed a nomogram based on the training set and integrated the PTPRS and other clinical features of patients using the R package “rms” and the performance of the nomogram was validated and calibrated using DCA, and time-independent ROC analysis in the training, validation and metaX cohorts.

### Statistical analysis

Group differences were evaluated using Mann–Whitney U test. Correlations between variables were analyzed with Pearson's or Spearman’s correlation analysis as appropriate. Outcomes with P < 0.05 were defined to be statistically significant in comparisons between groups. The R (version: 4.1.3) was used for data processing and statistical analyses.

### Ethics approval and consent to participate

The study was conducted in accordance with the Declaration of Helsinki, and approved by Medical Ethics Committee, Zhongnan Hospital, Wuhan University.

### Consent for publication

This research project has applied for exemption of patient informed consent to Medical Ethics Committee, Zhongnan Hospital, Wuhan University.

## Conclusions

In the present study, we demonstrated that the role of PTPNs in cancer may be related to mediating cell cycle related pathways, confirmed differential expression of PTPN2 at the clinical level between AML patients and normal subjects, indicated that AML may be a promising candidate for PTPN2 suppression immunotherapy, and constructed a nomogram for risk assessment in AML.

### Supplementary Information

Supplementary Information.

## Data Availability

All database generated/analyzed for this study are included/have their accession numbers included in the article.

## References

[1] Grimwade D, Ivey A, Huntly BJ (2016). Molecular landscape of acute myeloid leukemia in younger adults and its clinical relevance. Blood.

[2] Kayser S, Levis MJ (2022). Updates on targeted therapies for acute myeloid leukaemia. Br. J. Haematol..

[3] Newell LF, Cook RJ (2021). Advances in acute myeloid leukemia. BMJ.

[4] Lewis DR, Siembida EJ, Seibel NL, Smith AW, Mariotto AB (2021). Survival outcomes for cancer types with the highest death rates for adolescents and young adults, 1975–2016. Cancer-Am. Cancer Soc..

[5] Tang X, Qi C, Zhou H, Liu Y (2022). Critical roles of PTPN family members regulated by non-coding RNAs in tumorigenesis and immunotherapy. Front. Oncol..

[6] Abdel-Magid AF (2022). The inhibitors of Protein Tyrosine Phosphatase Nonreceptor Type 2 (PTPN2) as Potential Enhancers of Cancer Immunotherapy and Type 1 (PTPN1) as treatment of metabolic diseases. ACS Med. Chem. Lett..

[7] Li B, Yu L, Gao L (2022). Cancer classification based on multiple dimensions: SNV patterns. Comput. Biol. Med..

[8] Kuiper RP, Ligtenberg MJ, Hoogerbrugge N, Geurts VKA (2010). Germline copy number variation and cancer risk. Curr. Opin. Genet. Dev..

[9] Brennan K, Flanagan JM (2012). Epigenetic epidemiology for cancer risk: Harnessing germline epigenetic variation. Methods Mol. Biol..

[10] Manguso RT (2017). In vivo CRISPR screening identifies Ptpn2 as a cancer immunotherapy target. Nature.

[11] Fishel R (2015). Mismatch repair. J. Biol. Chem..

[12] Dembic Z (2020). Antitumor drugs and their targets. Molecules.

[13] Chen D, Zhang X, Li Z, Zhu B (2021). Metabolic regulatory crosstalk between tumor microenvironment and tumor-associated macrophages. Theranostics.

[14] Le Sommer S (2018). Deficiency in protein tyrosine phosphatase PTP1B shortens lifespan and leads to development of acute leukemia. Cancer Res..

[15] Fobare S (2022). Molecular, clinical, and prognostic implications of PTPN11 mutations in acute myeloid leukemia. Blood Adv..

[16] Duval R (2019). Benzoquinone, a leukemogenic metabolite of benzene, catalytically inhibits the protein tyrosine phosphatase PTPN2 and alters STAT1 signaling. J. Biol. Chem..

[17] Sharma B (2020). Recent advance on PTP1B inhibitors and their biomedical applications. Eur. J. Med. Chem..

[18] Liang S (2023). A small molecule inhibitor of PTP1B and PTPN2 enhances T cell anti-tumor immunity. Nat. Commun..

[19] Fujimura A (2019). PTPN3 expressed in activated T lymphocytes is a candidate for a non-antibody-type immune checkpoint inhibitor. Cancer Immunol. Immunother..

[20] Eswaran J (2006). Crystal structures and inhibitor identification for PTPN5, PTPRR and PTPN7: A family of human MAPK-specific protein tyrosine phosphatases. Biochem. J..

[21] Ahn D (2022). Ethyl gallate dual-targeting PTPN6 and PPARgamma shows anti-diabetic and anti-obese effects. Int. J. Mol. Sci..

[22] Liu Q, Qu J, Zhao M, Xu Q, Sun Y (2020). Targeting SHP2 as a promising strategy for cancer immunotherapy. Pharmacol. Res..

[23] Liu M, Gao S, Elhassan RM, Hou X, Fang H (2021). Strategies to overcome drug resistance using SHP2 inhibitors. Acta Pharm. Sin. B.

[24] Yang CF (2020). Targeting protein tyrosine phosphatase PTP-PEST (PTPN12) for therapeutic intervention in acute myocardial infarction. Cardiovasc. Res..

[25] Lee C, Rhee I (2019). Important roles of protein tyrosine phosphatase PTPN12 in tumor progression. Pharmacol. Res..

[26] Bertagnin C (2023). A small molecule targeting the interaction between human papillomavirus E7 oncoprotein and cellular phosphatase PTPN14 exerts antitumoral activity in cervical cancer cells. Cancer Lett..

[27] Long Q (2020). PTPN13 acts as a tumor suppressor in clear cell renal cell carcinoma by inactivating akt signaling. Exp. Cell Res..

[28] Lin G, Aranda V, Muthuswamy SK, Tonks NK (2011). Identification of PTPN23 as a novel regulator of cell invasion in mammary epithelial cells from a loss-of-function screen of the 'PTP-ome'. Genes Dev..

[29] Hoover AC (2009). Impaired PTPN13 phosphatase activity in spontaneous or HPV-induced squamous cell carcinomas potentiates oncogene signaling through the MAP kinase pathway. Oncogene.

[30] Jassim BA, Lin J, Zhang ZY (2022). PTPN22: Structure, function, and developments in inhibitor discovery with applications for immunotherapy. Expert Opin. Drug Discov..

[31] Dobosz P, Dzieciatkowski T (2019). The intriguing history of cancer immunotherapy. Front. Immunol..

[32] Kansara RR, Speziali C (2020). Immunotherapy in hematologic malignancies. Curr. Oncol..

[33] Kanumuri R, Kumar PS, Burns SS, Ramdas B, Kapur R (2022). Targeting SHP2 phosphatase in hematological malignancies. Expert Opin. Ther. Targets.

[34] Wu L (2022). Machine learning methods, databases and tools for drug combination prediction. Brief. Bioinform..

[35] Tomczak K, Czerwinska P, Wiznerowicz M (2015). The Cancer Genome Atlas (TCGA): An immeasurable source of knowledge. Contemp. Oncol..

[36] Goldman MJ (2020). Visualizing and interpreting cancer genomics data via the Xena platform. Nat. Biotechnol..

[37] Ma X (2018). Pan-cancer genome and transcriptome analyses of 1,699 paediatric leukaemias and solid tumours. Nature.

[38] Tyner JW (2018). Functional genomic landscape of acute myeloid leukaemia. Nature.

[39] Mermel CH (2011). GISTIC2.0 facilitates sensitive and confident localization of the targets of focal somatic copy-number alteration in human cancers. Genome Biol..

[40] Mayakonda A, Lin DC, Assenov Y, Plass C, Koeffler HP (2018). Maftools: Efficient and comprehensive analysis of somatic variants in cancer. Genome Res..

[41] Tomasson MH (2008). Somatic mutations and germline sequence variants in the expressed tyrosine kinase genes of patients with de novo acute myeloid leukemia. Blood.

[42] Metzeler KH (2008). An 86-probe-set gene-expression signature predicts survival in cytogenetically normal acute myeloid leukemia. Blood.

[43] Gaidzik VI (2011). RUNX1 mutations in acute myeloid leukemia: Results from a comprehensive genetic and clinical analysis from the AML Study Group. J. Clin. Oncol..

[44] Gaidzik VI (2012). TET2 mutations in acute myeloid leukemia (AML): Results from a comprehensive genetic and clinical analysis of the AML Study Group. J. Clin. Oncol..

[45] Li Z (2013). Identification of a 24-gene prognostic signature that improves the european leukemianet risk classification of acute myeloid leukemia: An international collaborative study. J. Clin. Oncol..

[46] Chuang MK (2015). An mRNA expression signature for prognostication in de novo acute myeloid leukemia patients with normal karyotype. Oncotarget.

[47] Herold T (2018). A 29-gene and cytogenetic score for the prediction of resistance to induction treatment in acute myeloid leukemia. Haematologica.

[48] Clough E, Barrett T (2016). The gene expression omnibus database. Methods Mol. Biol..

[49] Wu C, Jin X, Tsueng G, Afrasiabi C, Su AI (2016). BioGPS: Building your own mash-up of gene annotations and expression profiles. Nucleic Acids Res..

[50] The Genotype-Tissue Expression (GTEx) Project. *Nat. Genet.* **45**, 580–585 (2013). 10.1038/ng.2653 PMC4010069 23715323

[51] Ghandi M (2019). Next-generation characterization of the cancer cell line encyclopedia. Nature.

[52] Ritchie ME (2015). Limma powers differential expression analyses for RNA-sequencing and microarray studies. Nucleic Acids Res..

[53] Rhodes DR (2004). ONCOMINE: A cancer microarray database and integrated data-mining platform. Neoplasia.

[54] Li, T. et al. TIMER2.0 for analysis of tumor-infiltrating immune cells. *Nucleic Acids Res.* **48**, W509–W514 (2020). 10.1093/nar/gkaa407 PMC7319575 32442275

[55] Ellis MJ (2013). Connecting genomic alterations to cancer biology with proteomics: The NCI clinical proteomic tumor analysis consortium. Cancer Discov..

[56] Chandrashekar DS (2017). UALCAN: A portal for facilitating tumor subgroup gene expression and survival analyses. Neoplasia.

[57] Colwill K, Graslund S (2011). A roadmap to generate renewable protein binders to the human proteome. Nat. Methods.

[58] Rouillard AD (2016). The harmonizome: A collection of processed datasets gathered to serve and mine knowledge about genes and proteins. Database.

[59] Yuan H (2019). CancerSEA: A cancer single-cell state Atlas. Nucleic Acids Res..

[60] Sun D (2021). TISCH: A comprehensive web resource enabling interactive single-cell transcriptome visualization of tumor microenvironment. Nucleic Acids Res..

[61] Cerami E (2012). The cBio cancer genomics portal: An open platform for exploring multidimensional cancer genomics data. Cancer Discov..

[62] Ochoa D (2021). Open targets platform: Supporting systematic drug-target identification and prioritisation. Nucleic Acids Res..

[63] Liu CJ (2018). GSCALite: A web server for gene set cancer analysis. Bioinformatics.

[64] Shen W (2022). Sangerbox: A comprehensive, interaction-friendly clinical bioinformatics analysis platform. iMeta..

[65] Bonneville R (2017). Landscape of microsatellite instability across 39 cancer types. JCO Precis. Oncol..

[66] Thorsson V (2018). The immune landscape of cancer. Immunity.

[67] Malta TM (2018). Machine learning identifies stemness features associated with oncogenic dedifferentiation. Cell.

[68] Subramanian A (2005). Gene set enrichment analysis: A knowledge-based approach for interpreting genome-wide expression profiles. Proc. Natl. Acad. Sci. USA.

[69] Liberzon A (2015). The Molecular Signatures Database (MSigDB) hallmark gene set collection. Cell Syst..

[70] Sturm G, Finotello F, List M (2020). Immunedeconv: An R package for unified access to computational methods for estimating immune cell fractions from bulk RNA-sequencing data. Methods Mol. Biol..

[71] Yoshihara K (2013). Inferring tumour purity and stromal and immune cell admixture from expression data. Nat. Commun..

[72] Jiang P (2018). Signatures of T cell dysfunction and exclusion predict cancer immunotherapy response. Nat. Med..

[73] Zeng Z (2022). TISMO: Syngeneic mouse tumor database to model tumor immunity and immunotherapy response. Nucleic Acids Res..

[74] Zeng D (2021). IOBR: Multi-omics immuno-oncology biological research to decode tumor microenvironment and signatures. Front. Immunol..

[75] Kanehisa M, Furumichi M, Sato Y, Ishiguro-Watanabe M, Tanabe M (2021). KEGG: Integrating viruses and cellular organisms. Nucleic Acids Res..

[76] Resource TGO (2019). 20 years and Still GOing Strong. Nucleic Acids Res..

[77] Yang W (2013). Genomics of Drug Sensitivity in Cancer (GDSC): A resource for therapeutic biomarker discovery in cancer cells. Nucleic Acids Res..

[78] Maeser D, Gruener RF, Huang RS (2021). OncoPredict: An R package for predicting in vivo or cancer patient drug response and biomarkers from cell line screening data. Brief. Bioinform..

[79] Seashore-Ludlow B (2015). Harnessing connectivity in a large-scale small-molecule sensitivity dataset. Cancer Discov..

[80] Corsello SM (2020). Discovering the anti-cancer potential of non-oncology drugs by systematic viability profiling. Nat. Cancer.

[81] Yang C (2021). Prognosis and personalized treatment prediction in TP53-mutant hepatocellular carcinoma: An in silico strategy towards precision oncology. Brief. Bioinform..

[82] Geeleher P, Cox N, Huang RS (2014). PRRophetic: An R package for prediction of clinical chemotherapeutic response from tumor gene expression levels. PLoS ONE.

[83] Subramanian A (2017). A next generation connectivity map: L1000 Platform and the first 1,000,000 profiles. Cell.

[84] Yang C (2022). A survey of optimal strategy for signature-based drug repositioning and an application to liver cancer. ELife.

[85] Luna, A. et al. CellMiner Cross-Database (CellMinerCDB) Version 1.2: Exploration of patient-derived cancer cell line pharmacogenomics. *Nucleic Acids Res.* **49**, D1083–D1093 (2021). 10.1093/nar/gkaa968 PMC7779001 33196823

[86] Tibshirani R (1997). The Lasso Method for variable selection in the Cox model. Stat. Med..

[87] Engebretsen S, Bohlin J (2019). Statistical predictions with Glmnet. Clin. Epigenet..

[88] Love MI, Huber W, Anders S (2014). Moderated estimation of fold change and dispersion for RNA-seq data with DESeq2. Genome Biol..

[89] Wu, T. et al. ClusterProfiler 4.0: A universal enrichment tool for interpreting omics data. *Innovation* **2**, 100141 (2021). 10.1016/j.xinn.2021.100141 PMC8454663 34557778

[90] Walter W, Sanchez-Cabo F, Ricote M (2015). GOplot: An R package for visually combining expression data with functional analysis. Bioinformatics.

[91] Mogensen UB, Ishwaran H, Gerds TA (2012). Evaluating random forests for survival analysis using prediction error curves. J. Stat. Softw..

[92] Li F (2022). Construction of a solid Cox model for AML patients based on multiomics bioinformatic analysis. Front. Oncol..

[93] Zhang J (2022). GPX1-associated prognostic signature predicts poor survival in patients with acute myeloid leukemia and involves in immunosuppression. Biochim. Biophys. Acta Mol. Basis Dis..

[94] Jiang N (2022). Identification of a mitochondria-related gene signature to predict the prognosis in AML. Front Oncol..

[95] Zhu R, Tao H, Lin W, Tang L, Hu Y (2020). Identification of an immune-related gene signature based on immunogenomic landscape analysis to predict the prognosis of adult acute myeloid leukemia patients. Front. Oncol..

[96] Dong C, Zhang N, Zhang L (2021). The multi-omic prognostic model of oxidative stress-related genes in acute myeloid leukemia. Front. Genet..

[97] Jeschke J (2017). DNA methylation-based immune response signature improves patient diagnosis in multiple cancers. J. Clin. Invest..

