# Supplementary material for: Pan-cancer analysis revealing that PTPN2 is an indicator of risk stratification for acute myeloid leukemia
Source: Sci Rep. 2023 Oct 26;13:18372. doi: 10.1038/s41598-023-44892-z (PMC10603079; doi:10.1038/s41598-023-44892-z)
Supplement: Supplementary file 1 — Supplementary Information. [file 41598_2023_44892_MOESM1_ESM.docx]

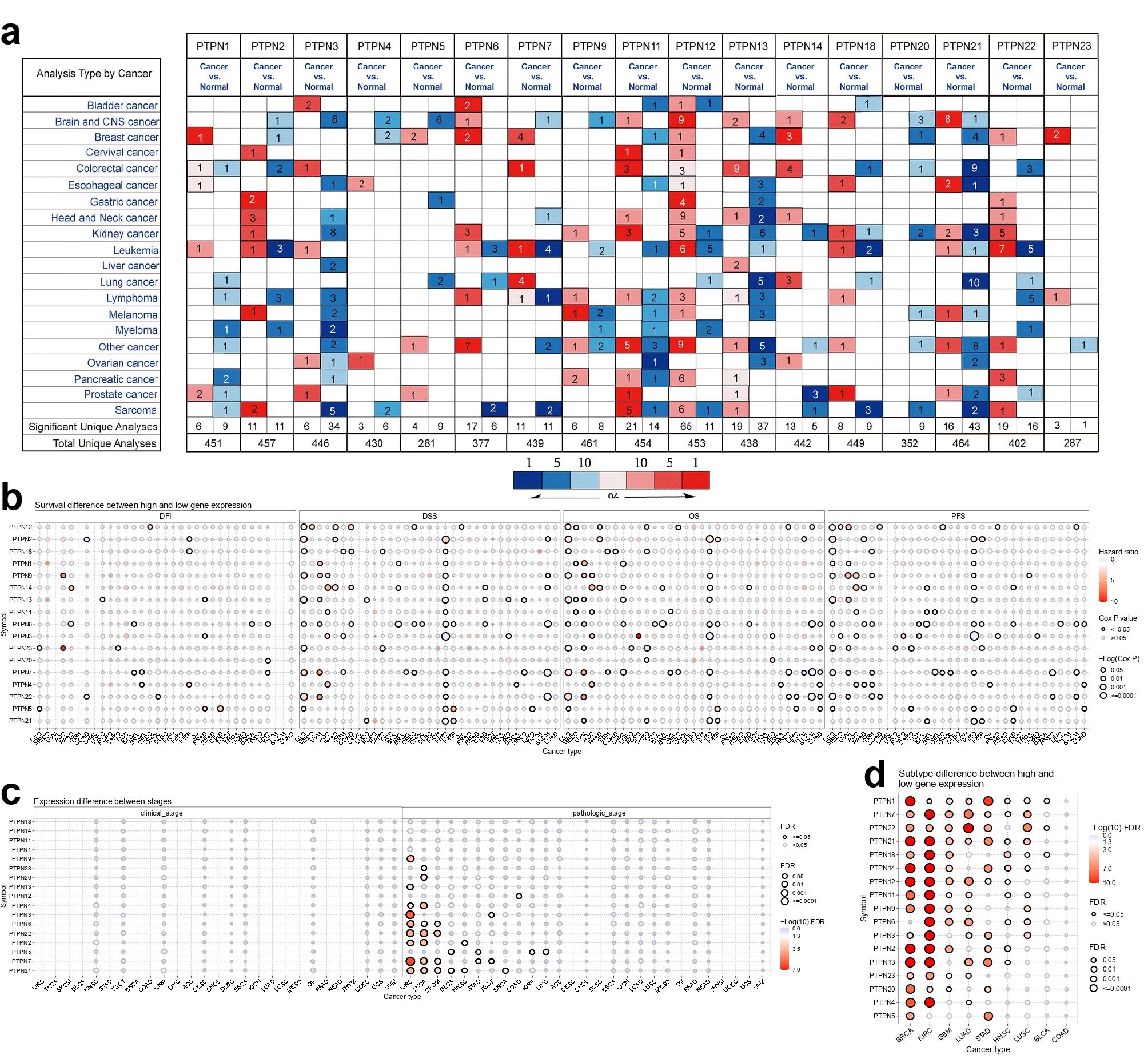


**Fig. S1.** (a) Differential expression of PTPNs in human Oncomine datasets. (b-d) Differential expression of PTPNs among clinical stage, pathologic stage, and subtypes.


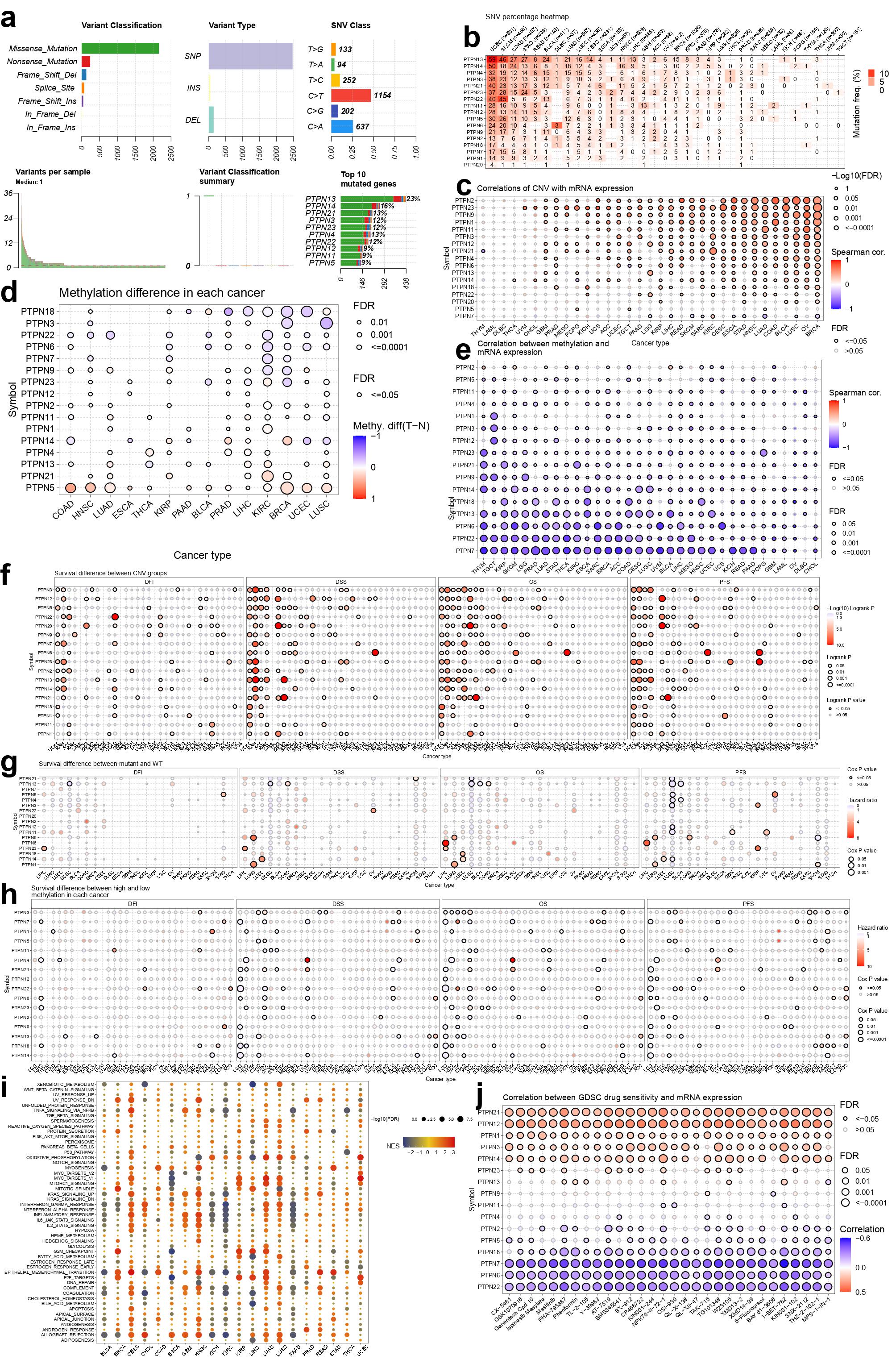


**Fig. S2.** (a) Summary of variants for PTPNs in pan-cancer. (b) The frequency of mutations for PTPNs in pan-cancer. (c) The correlation between PTPNs expression and CNV. (d) The difference of PTPNs methylation between tumor and normal samples in pan-cancer. (e) The correlation between PTPNs expression and methylation. (f-h) Prognostic associations of CNV, SNV, and methylation in PTPNs. (i) Enrichment analysis for metabolism pathway and cancer signaling between high and low enrichment scores of PTPNs. (j) Correlation between PTPNs expression and drug IC50.


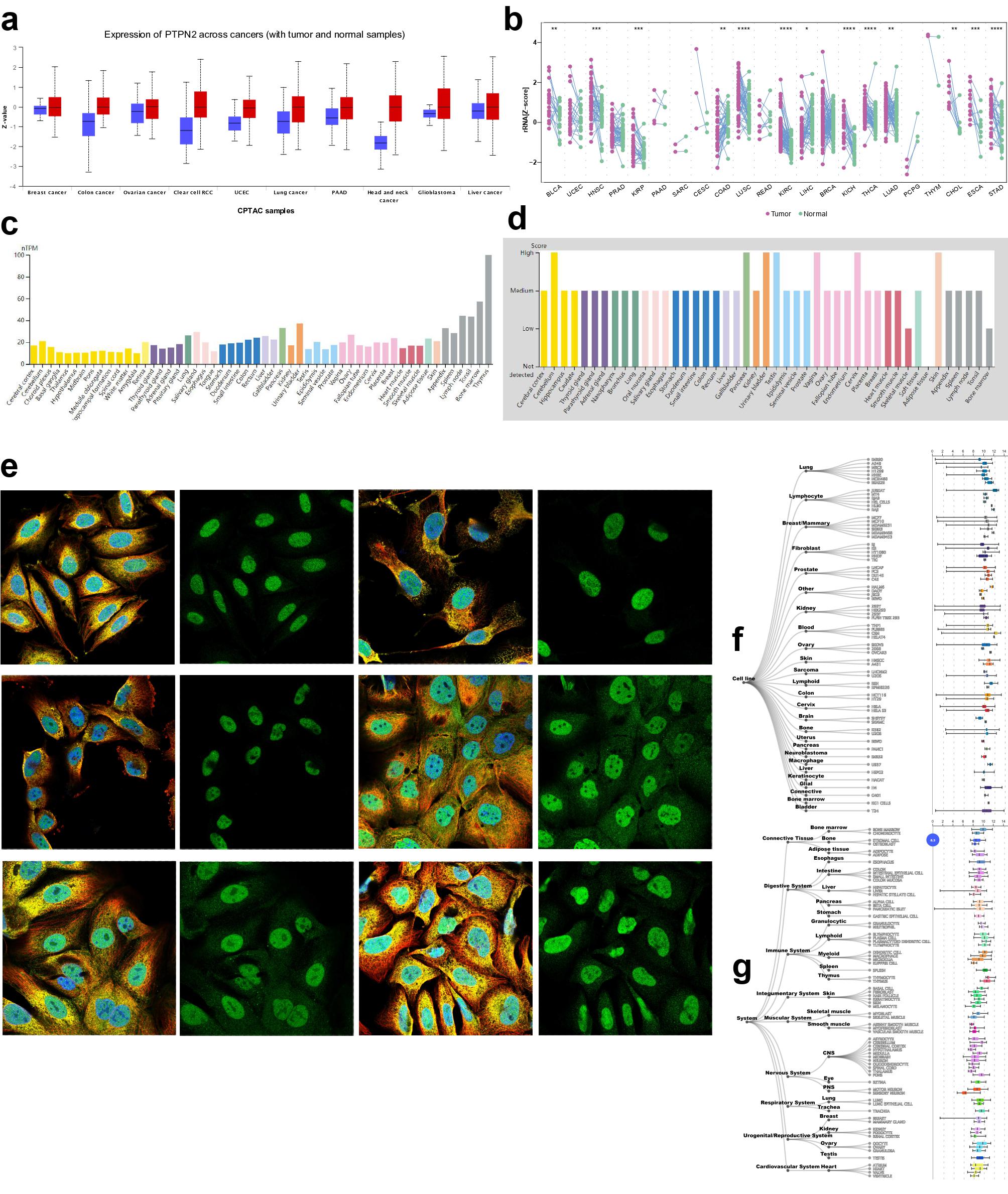


**Fig. S3.** (a) Expression analysis of PTPN2 protein in pan-cancer. (b) Differential gene expression analysis of PTPN2 in the paired samples. (c-d) RNA and protein expression score of PTPN2. (e) Subcellular localization of PTPN2 in different cancer cell lines. (f-g) Expression of PTPN2 in different cell lines and tissues.


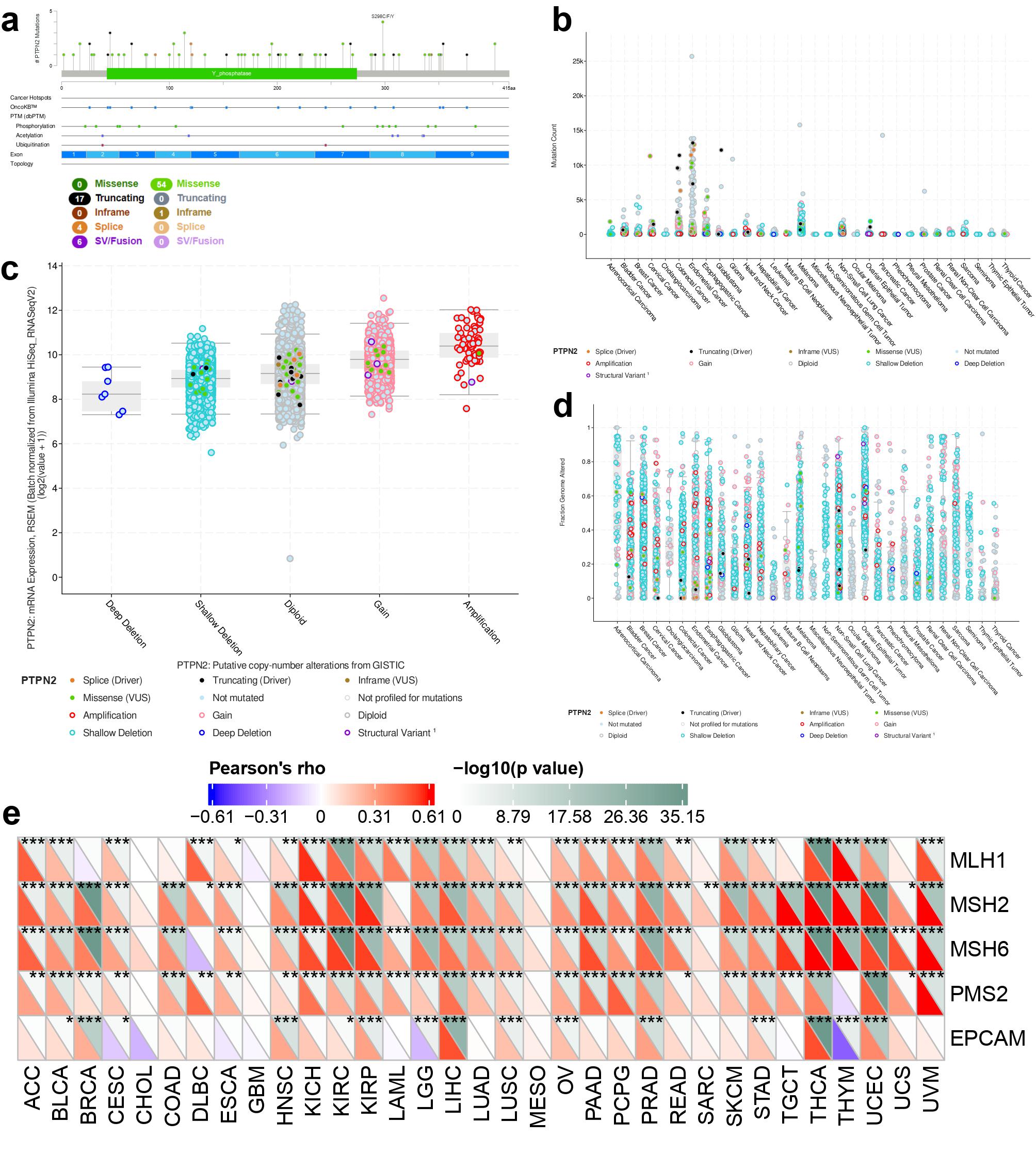


**Fig. S4.** (a) lollipop of PTPN2. (b) Mutation count of PTPN2. (c)PTPN2 expression in different types of CNV. (d) FGA of PTPN2. (e) Correlation between PTPN2 and MMRs related genes.


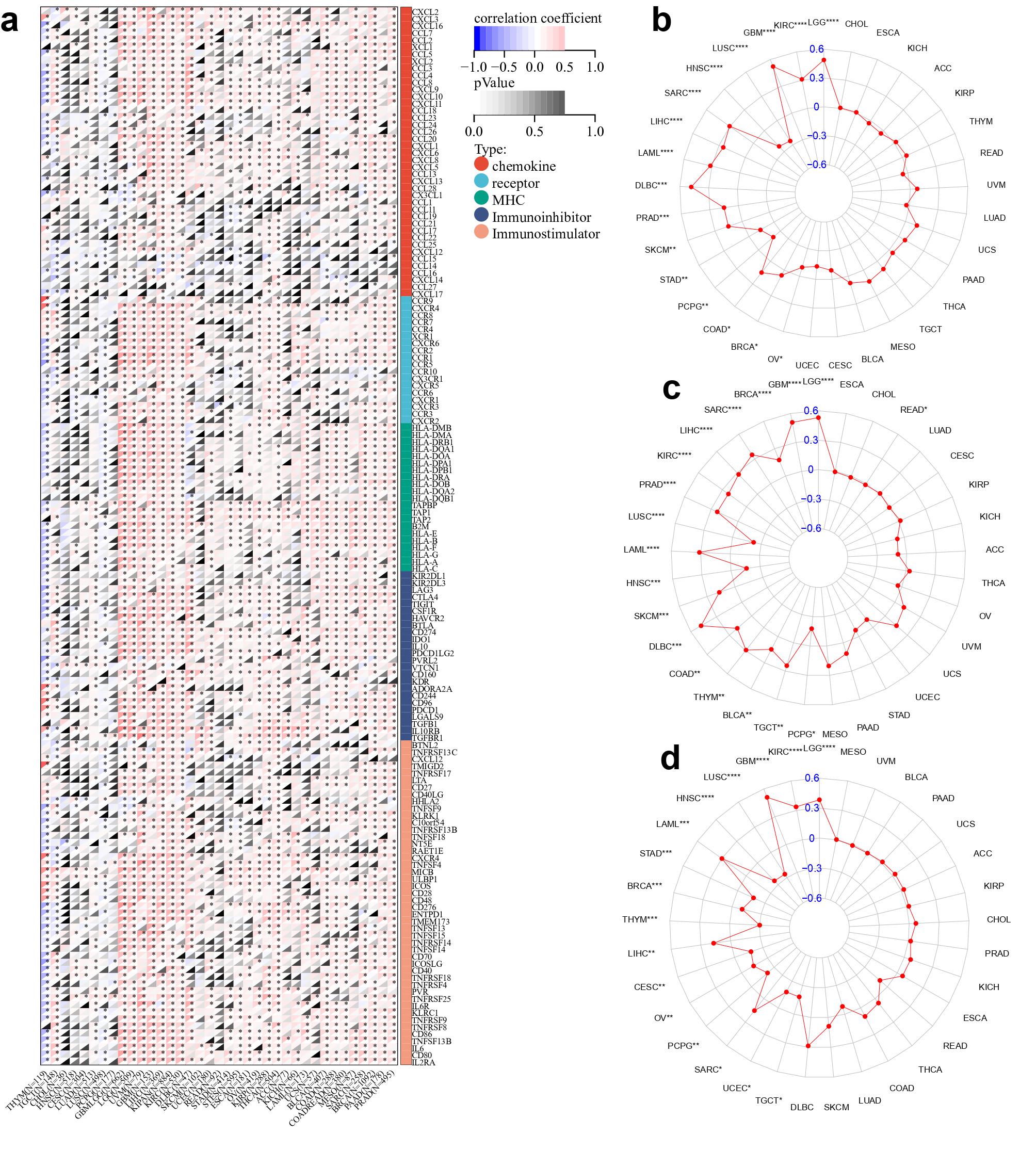


**Fig. S5.** (a) Correlation between PTPN2 and immunomodulatory genes. (b-d) Correlation of PTPN2 expression with ESTIMATE score, Immune score, and Stromal score.


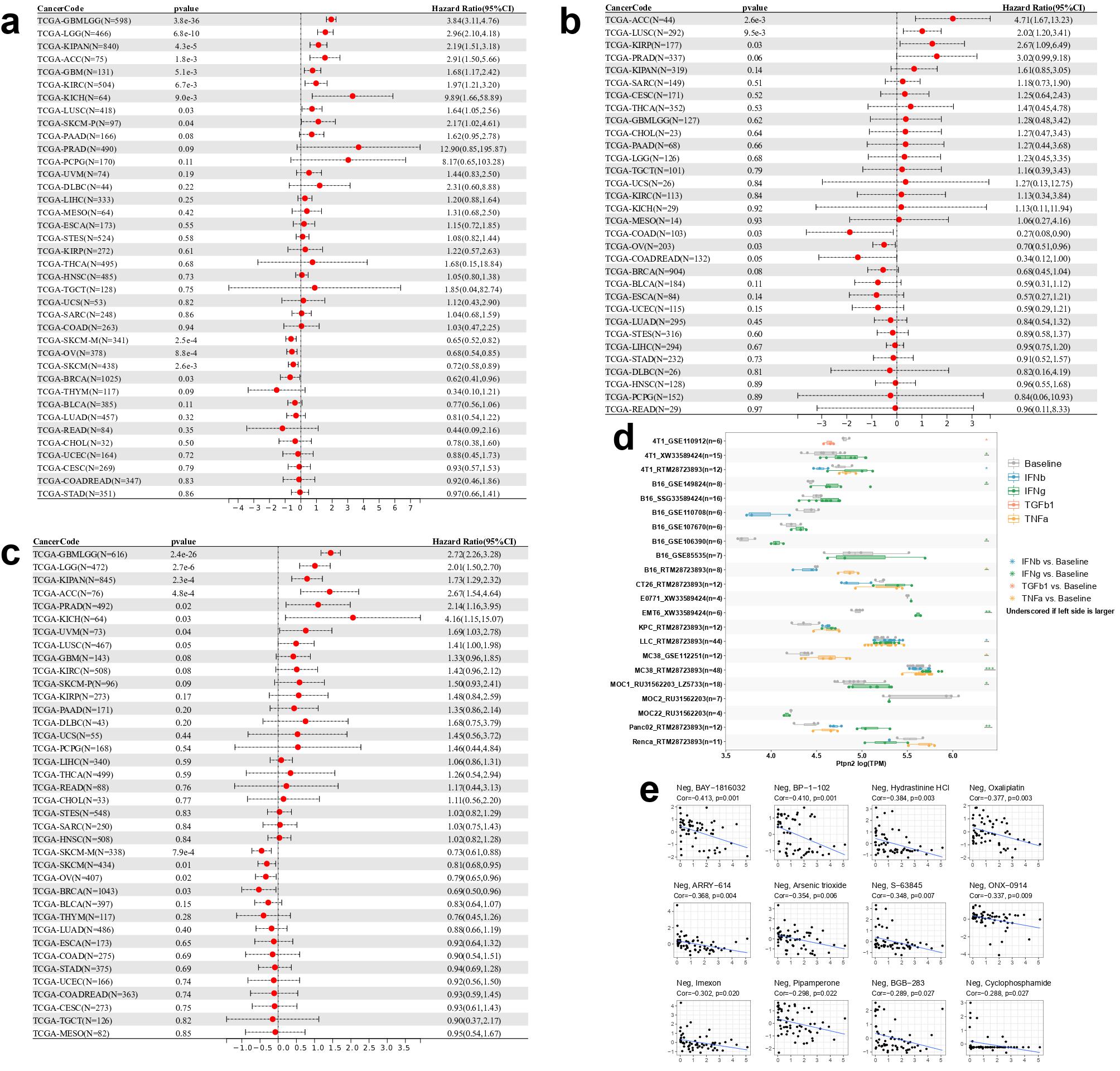


**Fig. S6.** Correlation between PTPN2 expression and (a) disease-specific survival, (b) disease-free interval, and (c) progression-free interval. (d) A Comparison of PTPN2 expression before and after ICB treatments across different tumor models in vitro. (e) The top 12 drugs negatively correlated with PTPN2 expression in the CellMiner database.


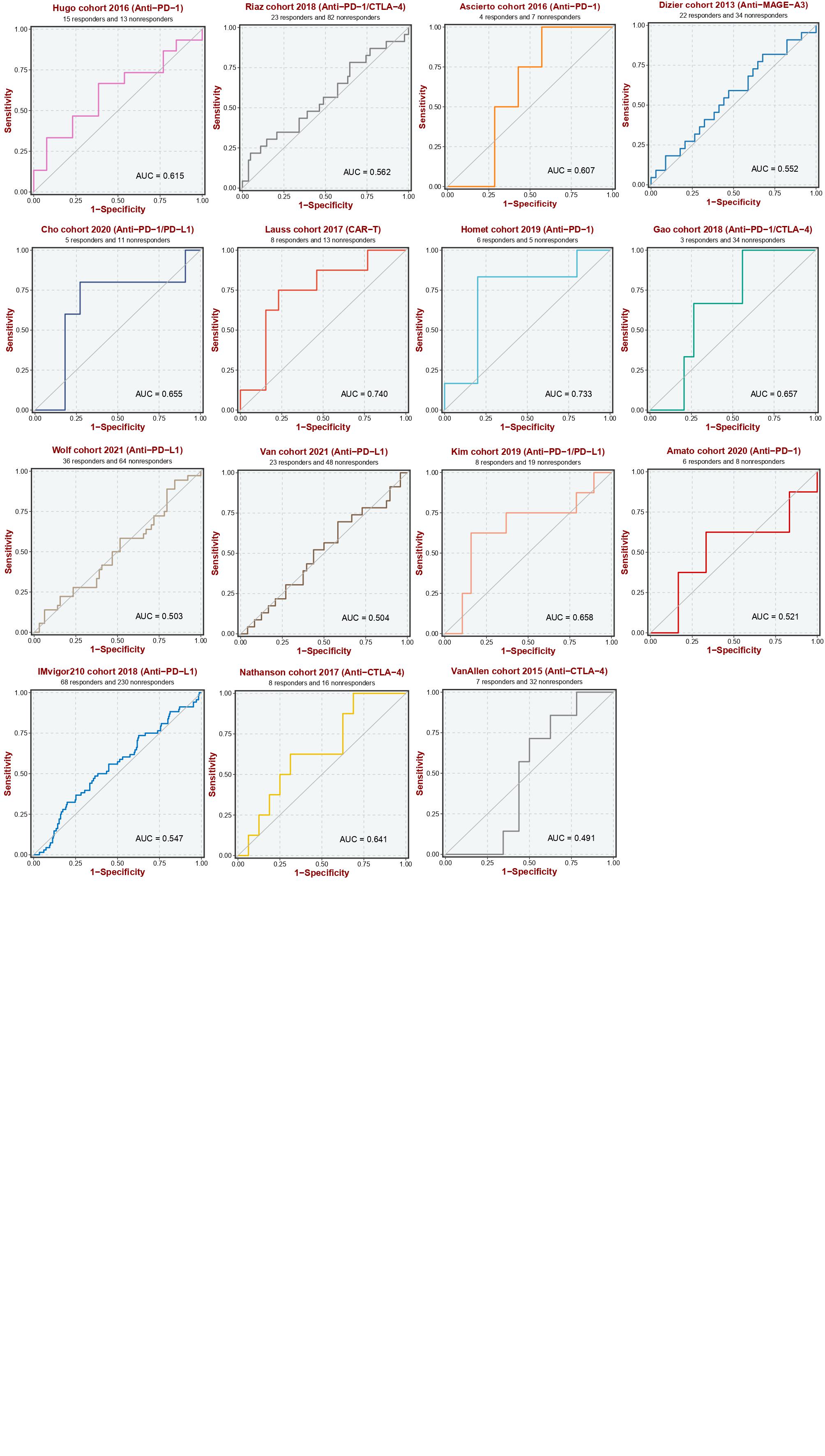


**Fig. S7.** The predictive value of PTPN2 to predict survival prognosis in patients in immunotherapy cohorts.


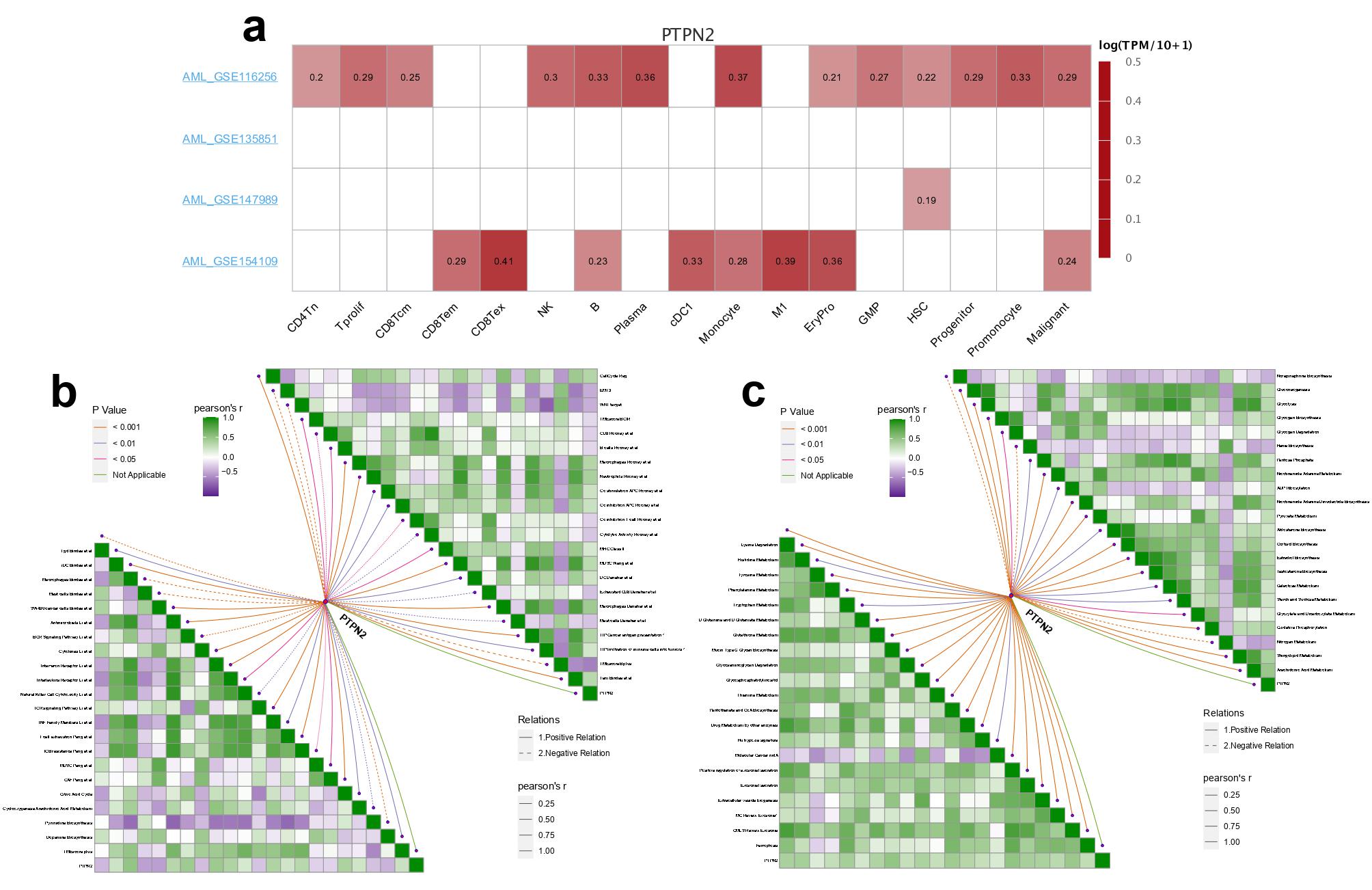


**Fig. S8.** (a) The expression of PTPN2 in different cell types. (b-c) Correlation between PTPN2 expression and cancer microenvironment-related signatures in TARGT-AML.


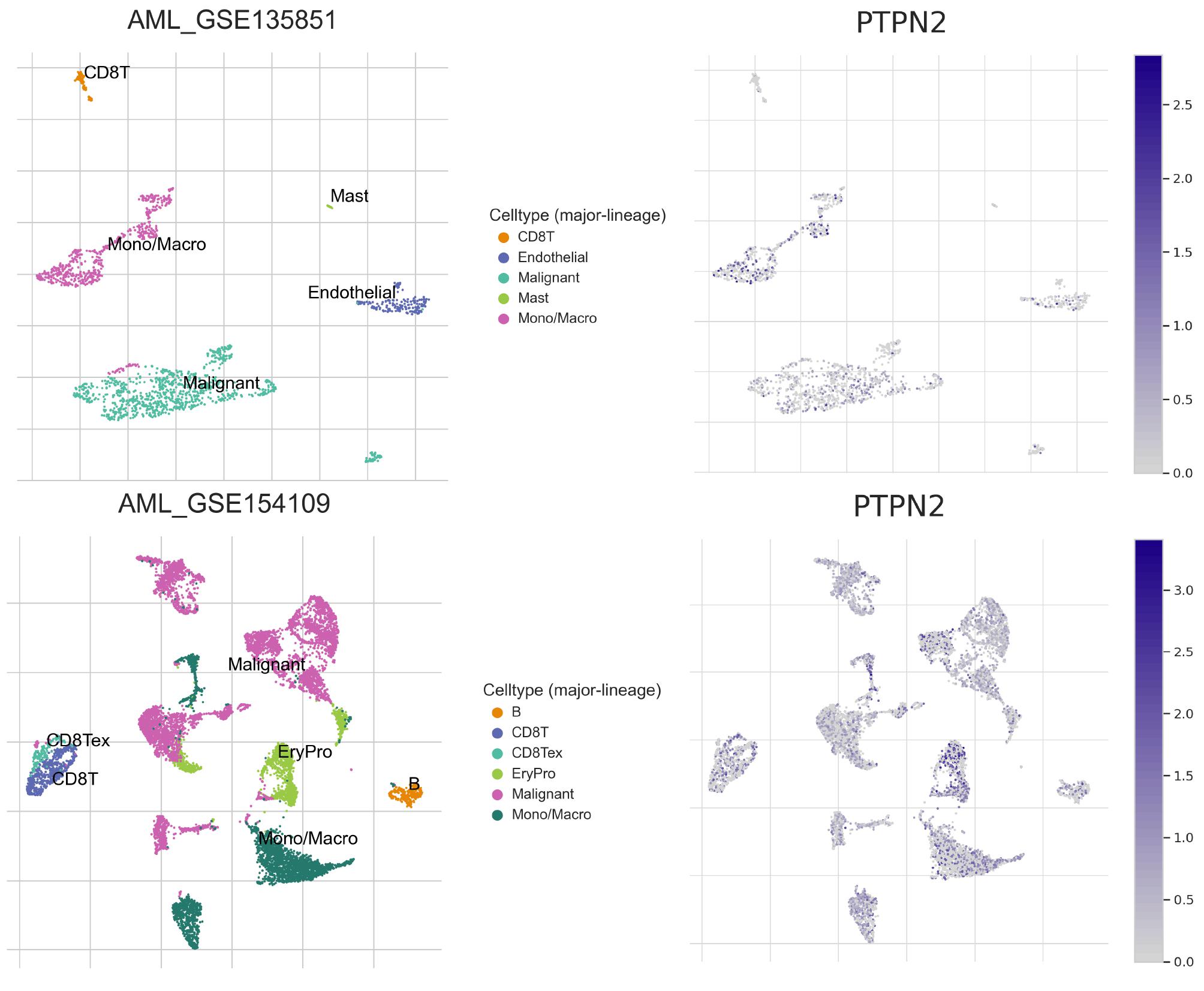


**Fig. S9.** The expression of PTPN2 in single-cell RNA sequencing cohort.


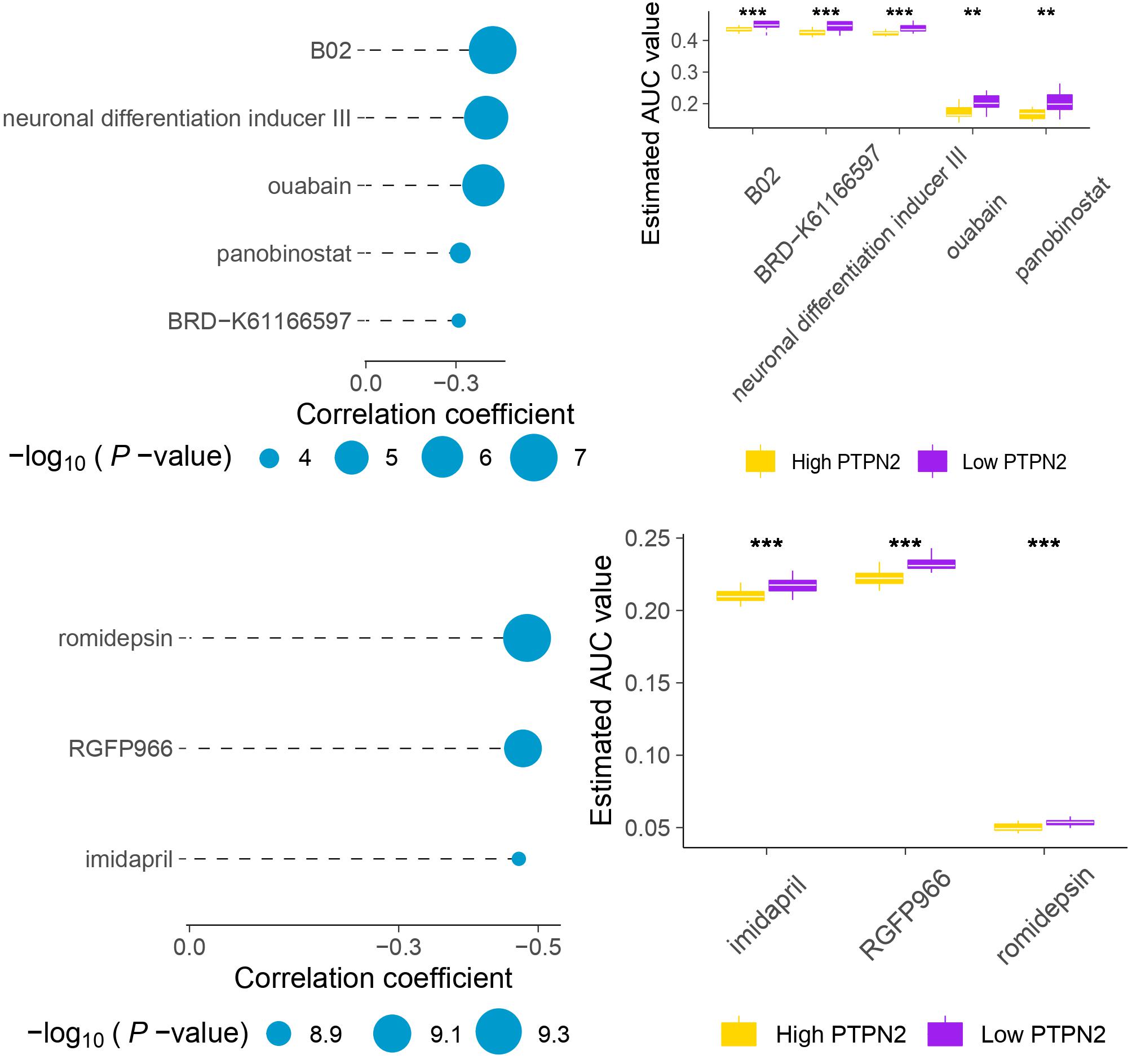


**Fig. S10.** Drug screening in patients with low PTPN2 expression in CTRP and PRISM.


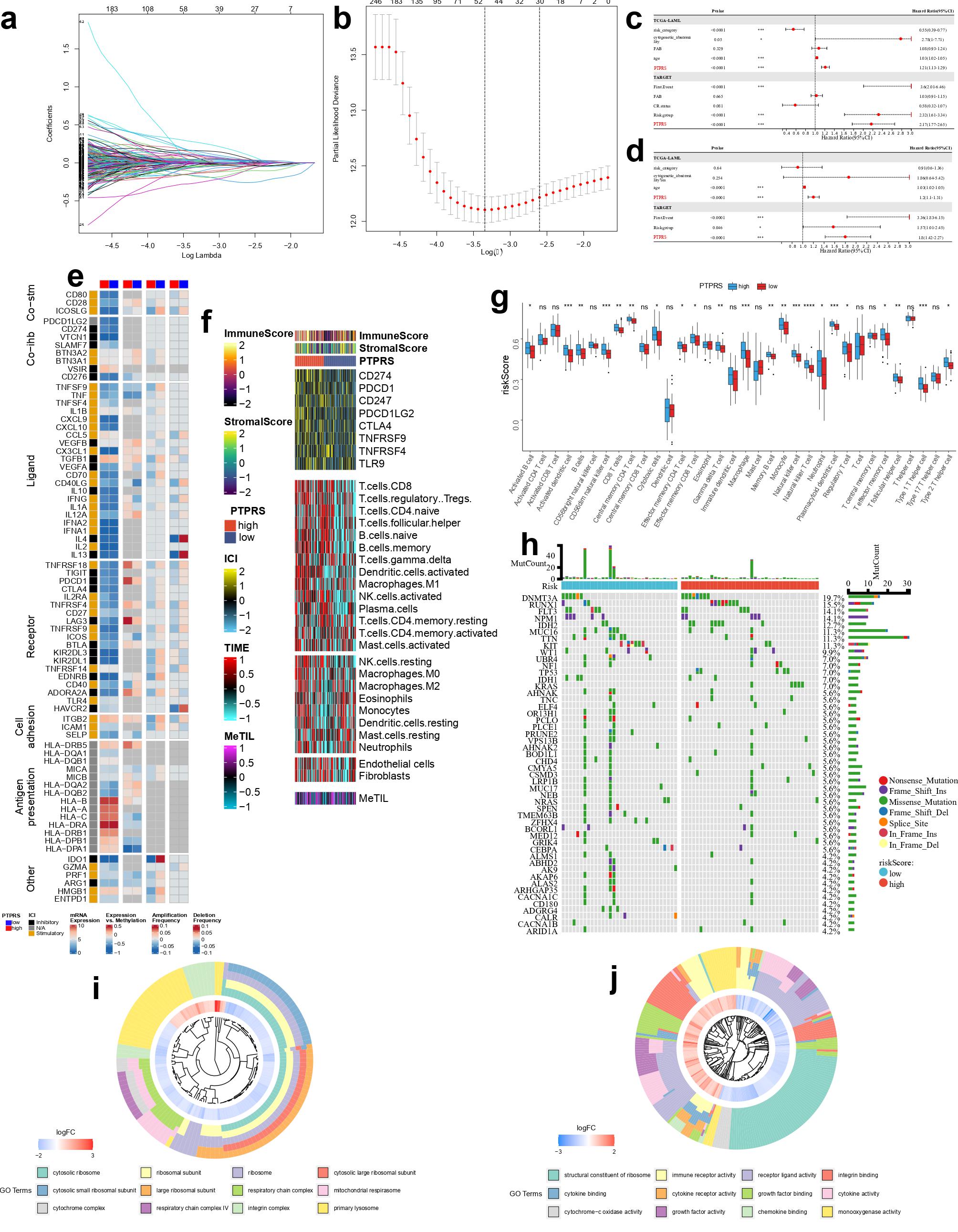


**Fig. S11.** (a) LASSO coefficient distribution of 826 prognostic genes in the training cohort. Coefficient profiles are drawn according to logarithmic (Lambda) sequences. (b) Cross-validation of turning parameters selected by minimum criteria in LASSO regression models. Use the minimum criterion to draw two dotted lines at the optimal value. The best genes (30 in total) with the best recognition ability were selected. (c) Univariate and (d) multivariate Cox regression analysis in TCGA-LAML and TARGET-AML cohort. (e) From left to right: mRNA expression (median normalized expression level); Expression and methylation (correlation between gene expression and DNA methylated beta-value); Amplification frequency (the difference between the high-risk and low-risk group for which specific subtypes of immunomodulators were amplified and the amplification fraction in all samples); Deletion frequency of 75 immunomodulatory genes was analyzed by PTPRS. (f) From left to right: mRNA expression (median normalized expression level); expression and methylation (correlation between gene expression and DNA methylated beta-value); amplification frequency (the difference between the high-risk and low-risk group for which specific subtypes of immunomodulators were amplified and the amplification fraction in all samples); deletion frequency of 75 immunomodulatory genes was analyzed by PTPRS. (g) Differential expression of 27 survival related immune cell types in high-risk and low-risk group based on ssGSEA algorithm. (h) Differential expression of 27 survival related immune cell types in high-risk and low-risk group based on ssGSEA algorithm. (i-j) Functional and signaling pathway analysis of differential genes between high-risk and low-risk group in AML.


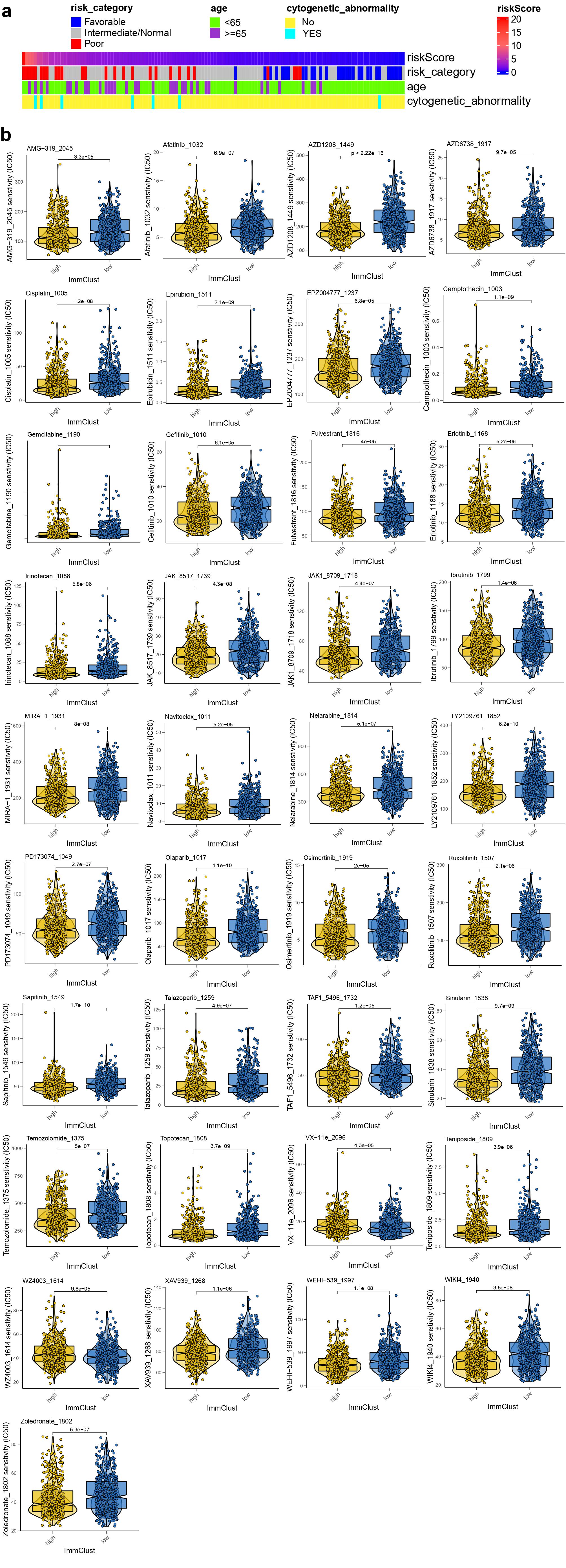


**Fig. S12.** (a) The heatmap for the association between clinicopathologic features and PTPRS. (b) The IC50 value of different tumor therapeutics in the high-risk and low-risk group.

Supplementary Table S1. Full names and abbreviations of all cancers

| Full name | Abbreviation |
| --- | --- |
| Adrenocortical carcinoma  Acute Lymphoblastic Leukemia  Bladder Urothelial Carcinoma  Breast invasive carcinoma  Cervical squamous cell carcinoma and endocervical adenocarcinoma  Cholangiocarcinoma  Chronic lymphocytic leukemia  Chronic myelocytic leukemia  Colon adenocarcinoma  Colon adenocarcinoma/Rectum adenocarcinoma Esophageal carcinoma  Lymphoid Neoplasm Diffuse Large B-cell Lymphoma  Ewing’s sarcoma  Esophageal carcinoma  Glioblastoma multiforme  Glioma  Head and Neck squamous cell carcinoma  Kidney Chromophobe  Pan-kidney cohort (KICH+KIRC+KIRP)  Kidney renal clear cell carcinoma  Kidney renal papillary cell carcinoma  Acute Myeloid Leukemia  Brain Lower Grade Glioma  Liver hepatocellular carcinoma  Lung adenocarcinoma  Lung squamous cell carcinoma  Medulloblastoma  Mesothelioma  Multiple Myeloma  Neuroblastoma  Non-small cell lung carcinoma  Ovarian serous cystadenocarcinoma  Pancreatic adenocarcinoma  Pheochromocytoma and Paraganglioma  Prostate adenocarcinoma  Rectum adenocarcinoma  Sarcoma  Small cell carcinoma of lung  Skin Cutaneous Melanoma  Stomach adenocarcinoma  Stomach and Esophageal carcinoma  Testicular Germ Cell Tumors  Thyroid carcinoma  Thymoma  Uterine Corpus Endometrial Carcinoma  Uterine Carcinosarcoma  Uveal Melanoma  High-Risk Wilms Tumor | ACC  ALL  BLCA  BRCA  CESC  CHOL  CLL  LCML  COAD  COADREAD  DLBC  ES  ESCA  GBM  GBMLGG  HNSC  KICH  KIPAN  KIRC  KIRP  LAML  LGG  LIHC  LUAD  LUSC  MB  MESO  MM  NB  NSC  OV  PAAD  PCPG  PRAD  READ  SARC  SCLC  SKCM  STAD  STES  TGCT  THCA  THYM  UCEC  UCS  UVM  WT |

Supplementary Table S2. The sequences of primers for quantitative real-time PCR assays.

| Gene | Sequence |
| --- | --- |
| PTPN2-F | ATCGAGCGGGAGTTCGA |
| PTPN2-R | TCTGGAAACTTGGCCACTC |
| GAPDH-F | CAGCCTCAAGATCATCAGCAA |
| GAPDH-R | TGTGGTCATGAGTCCTTCCA |

Supplementary Table S3. Gene in the prognostic classifier.

| Gene | LASSO coefficient |
| --- | --- |
| ETS2 | 0.152206159 |
| FGR | 0.160595683 |
| GSTK1 | -0.323989063 |
| NR4A2 | -0.078208265 |
| HOXA10 | 0.082900421 |
| MTHFR | 0.2605123 |
| ZFR | -0.30884948 |
| CCND3 | 0.229274358 |
| HYAL2 | -0.128095236 |
| LSM3 | 0.295255661 |
| MICALL2 | -0.130326596 |
| PACS2 | 0.445454662 |
| MTMR9 | 0.408594217 |
| RAB22A | -0.305984026 |
| CYP1B1 | 0.127854503 |
| GCH1 | 0.190644948 |
| AOAH | -0.185088463 |
| HCFC2 | 0.378472849 |
| ABCA1 | -0.125409636 |
| DNAJC15 | 0.39084415 |
| PDE8A | -0.508755306 |
| LY96 | -0.322871014 |
| TBR1 | -0.253813366 |
| GPD1 | 0.407917155 |
